# Reduced plasma levels of small HDL particles transporting fibrinolytic proteins in pulmonary arterial hypertension

**DOI:** 10.1136/thoraxjnl-2018-212144

**Published:** 2018-11-26

**Authors:** Lars Harbaum, Pavandeep Ghataorhe, John Wharton, Beatriz Jiménez, Luke S G Howard, J Simon R Gibbs, Jeremy K Nicholson, Christopher J Rhodes, Martin R Wilkins

**Affiliations:** 1 Department of Medicine, Imperial College London, London, UK; 2 Imperial Clinical Phenome Centre, Department of Surgery and Cancer, Imperial College London, London, UK; 3 National Heart and Lung Institute, Imperial College London, London, UK; 4 National Pulmonary Hypertension Service, Imperial College Healthcare Trust NHS, London, UK; 5 Division of Computational Systems Medicine, Department of Surgery and Cancer, and Centre for Digestive and Gut Health, Imperial College London, London, UK

**Keywords:** primary pulmonary hypertension

## Abstract

**Background:**

Aberrant lipoprotein metabolism has been implicated in experimental pulmonary hypertension, but the relevance to patients with pulmonary arterial hypertension (PAH) is inconclusive.

**Objective:**

To investigate the relationship between circulating lipoprotein subclasses and survival in patients with PAH.

**Methods:**

Using nuclear magnetic resonance spectroscopy, 105 discrete lipoproteins were measured in plasma samples from two cohorts of patients with idiopathic or heritable PAH. Data from 1124 plasma proteins were used to identify proteins linked to lipoprotein subclasses. The physical presence of proteins was confirmed in plasma lipoprotein subfractions separated by ultracentrifugation.

**Results:**

Plasma levels of three lipoproteins from the small high-density lipoprotein (HDL) subclass, termed HDL-4, were inversely related to survival in both the discovery (n=127) and validation (n=77) cohorts, independent of exercise capacity, comorbidities, treatment, N-terminal probrain natriuretic peptide, C reactive protein and the principal lipoprotein classes. The small HDL subclass rich in apolipoprotein A-2 content (HDL-4-Apo A-2) exhibited the most significant association with survival. None of the other lipoprotein classes, including principal lipoprotein classes HDL and low-density lipoprotein cholesterol, were prognostic. Three out of nine proteins identified to associate with HDL-4-Apo A-2 are involved in the regulation of fibrinolysis, namely, the plasmin regulator, alpha-2-antiplasmin, and two major components of the kallikrein–kinin pathway (coagulation factor XI and prekallikrein), and their physical presence in the HDL-4 subfraction was confirmed.

**Conclusion:**

Reduced plasma levels of small HDL particles transporting fibrinolytic proteins are associated with poor outcomes in patients with idiopathic and heritable PAH.

**Key messages**

**What is the key question?**

- What is the relationship between circulating lipoproteins levels and outcomes in patients with pulmonary arterial hypertension (PAH)?

**What is the bottom line?**

- Reduced levels of the smallest subclass of high-density lipoprotein (HDL) particle, termed HDL-4, are associated with high mortality in patients with idiopathic or heritable PAH. Particles of this small HDL subclass transport proteins controlling fibrinolysis, such as prekallikrein, a mechanism likely to be responsible for the association with clinical outcomes.

**Why read on?**

- Idiopathic or heritable PAH is a rare but fatal vascular disorder with poor patient outcomes. The involvement of lipoproteins, which are critical for vascular homoeostasis, has not been defined.

## Introduction

Idiopathic and heritable pulmonary arterial hypertension (PAH) is a proliferative vascular disorder that results in raised pulmonary vascular resistance, leading to right heart failure and premature death.^1^ It is characterised by obstructive vascular remodelling of pulmonary arteries and arterioles, perivascular inflammation and metabolic changes, including alterations in beta-oxidation and aerobic glycolysis.^2^ Genetic and metabolic studies support the clinical observation that PAH is a heterogeneous condition with variation in life expectancy and response to treatment.^3 4^

Low-density lipoproteins (LDL) are active participants in atherosclerotic vascular disease and constitute major drug targets; reduced levels are associated with a significant reduction in cardiovascular disease.^5^ Contrary to the benefits seen in atherosclerosis, treatment with statins has no significant effect on exercise capacity, cardiac output^6^ and mortality^7^ in PAH.

High-density lipoproteins (HDLs) facilitate cholesterol efflux from tissues and exhibit vasodilatory, anti-inflammatory, and endothelial protective properties, which would be expected to abrogate the development of pulmonary vascular remodelling.^8^ Indeed, a peptide mimetic of apolipoprotein (Apo) A-1, the major protein component of HDL, has been reported to reduce pulmonary hypertension in rodent models of this condition.^9 10^ On this basis, raised circulating HDL might be expected to be associated with improved survival in patients with PAH but studies to date have provided contrasting results.^11–14^

HDL lipoproteins are heterogeneous in size, structure and lipid/protein composition.^15^ Proton nuclear magnetic resonance (NMR) spectroscopy has been widely used in metabolic profiling of plasma samples,^16^ and recent advances have enabled quantitative analysis of multiple lipoprotein subclasses.^17^ We hypothesised that differences in the levels of lipoprotein subclasses, and in particular their associated proteome,^18–20^ may provide insight into the relationship between HDL and the natural history of PAH.

We used NMR spectroscopy to investigate two cohorts of patients with idiopathic and heritable PAH. We discovered and validated that circulating levels of the small HDL subclass, termed HDL-4, independently associated with patient survival. We identified nine proteins associated with small HDL-4 and directly confirmed the presence of fibrinolysis-linked proteins in this plasma subfraction. Enrichment and network analyses revealed the kallikrein–kinin pathway, prekallikrein in particular, as a likely biological mechanism linking HDL-4 to the progression of PAH.

## Methods

### Study cohorts and clinical measurements

We analysed two cohorts of patients with idiopathic or heritable PAH who attended the National Pulmonary Hypertension Service at Hammersmith Hospital, London, UK. The diagnosis of PAH was made using standard diagnostic criteria and internationally agreed guidelines.^21^ To rule out a post capillary component in patients with pulmonary artery wedge presseru >15 mm Hg but no other findings indicating left heart failure concomitant left ventricular end diastolic pressure was measured. The first (discovery) cohort comprised 127 consecutive patients with PAH recruited between 1 November 2011 and 13 August 2013. A distinct second (validation) cohort of 77 patients with PAH was recruited between 7 April 2003 and 2 April 2014 (patients recruited between 1 November 2011 and 13 August 2013 but whose samples failed for technical reasons in the first experiment were reanalysed in the second experiment). Patients in both cohorts were predominantly prevalent cases. The two cohorts of patients were censored on 1 June 2017. No patient was lost to follow-up and no patient was referred for lung transplantation.

Venous blood samples were drawn from the antecubital fossa and collected in ethylenediaminetetraacetic acid-Vacutainer tubes (BD, Oxford, UK) under non-fasting conditions during routine clinical appointment visits. Samples were held on ice, processed within 30 min and plasma was stored at −80°C until thawed for experiment. Aliquots were used for NMR, proteomic analysis and measurement of N-terminal pro-brain natriuretic peptide (NT-proBNP). All samples and data were obtained with informed written consent and local research ethics committee approval.

Clinical data including WHO functional class and 6 min walk distance (6MWD) were recorded on the date of sampling. Further clinical biochemical data including creatinine, bilirubin, HDL cholesterol, LDL cholesterol and C reactive protein (CRP), were recorded within 30 days of the sample date. The subgroup of patients with PAH and limited cardiovascular comorbidities  were defined by having less than three risk factors for left heart failure.^22^ The subgroup of patients with incident disease was defined by having been sampled within a period of 180 days from diagnosis.

### NMR spectroscopy

NMR data were acquired using a 600 MHz Avance III NMR Spectrometer (Bruker Biospin) following the protocol previously described.^23^ Lipoprotein analysis of the ^1^H NMR spectra was conducted using Bruker IVDr Lipoprotein Subclass Analysis (B.I.-LISA) developed by Bruker BioSpin GmbH, Germany, which defined the concentration of 105 distinct lipoproteins (online supplementary methods). Briefly, data from a training sample set, separated by density-gradient ultracentrifugation, was used to construct a linear regression model that used the ^1^H NMR spectra to predict the concentration of distinct lipoproteins subclasses in samples.^24^ The four principal lipoprotein classes (VLDL, IDL, LDL and HDL) were separated in up to six distinct subclasses, which were labelled numerically, starting with the lowest density.

10.1136/thoraxjnl-2018-212144.supp1 Supplementary file 1

### Plasma proteome measurements

Circulating plasma proteins were measured using SOMAscan V.3 (Somalogic, Boulder, Colorado, USA), which uses DNA-based aptamer reagents to define the levels of 1124 proteins.^25^

### Density-gradient ultracentrifugation

Ultracentrifugation was performed as previously described.^26^ Briefly, the background density of plasma samples from eight patients with PAH was adjusted to 1.125 kg/L by potassium bromide using the formula provided by Havel *et al*,^27^ and overlaid with aqueous solution of normal saline adjusted to the identical density. Ultracentrifugation was performed for 48 hours at 50 000 rpm using a fixed angle rotor (Type 70.1 Ti, Beckman Coulter, Brea, California, USA).

### Enzyme-linked immunosorbent assay

ELISAs were performed on whole plasma and plasma subfractions. To measure Apo A-1 levels, samples were diluted 1:20 000 and added 1:2 to the diluent (R&D Systems, #DAPA10, Minneapolis, Minnesota, USA). For prekallikrein measurements, samples were diluted 1:4000 (Abcam, #ab171015, Cambridge, UK) and for neuropilin-1, samples were diluted 1:200 and added to 1:1 to the diluent (R&D Systems, #DNRP10).

### Statistical analyses

Prior to analysis, NMR lipoprotein and plasma proteome data were transformed to Z-scores (by subtracting the mean and dividing by the SD) for ease of comparison. Plasma proteome data were log-transformed prior to Z-score transformation.

Survival analyses were performed using time from sampling to death/census. First, potential confounding factors, including patient demographics, comorbidities, treatment, renal and hepatic function, were assessed by separate Cox regression analysis and the three most significant factors (age, diuretic treatment and atrial fibrillation) were selected as covariates for the lipoprotein survival analysis (online supplementary table S1). Preserved renal function was defined as creatinine <75 µmol/L and liver function as bilirubin <21 µmol/L. Applying a backward-forward selection process to fit a multivariate Cox model (with p>0.05 as removal and p<0.05 as entry criterion) confirmed that these parameters provided independent prognostic information. Cox regression analysis was then used to identify prognostic lipoprotein subclasses individually, adjusting for these covariates. Subsequently, comparisons were also made against the principal lipoprotein classes (HDL cholesterol and LDL cholesterol), the inflammatory marker CRP, the 6WMD and the marker NT-proBNP to show the specificity and independence of the subclasses.

To account for multiple testing, we applied conservative Bonferroni corrected thresholds using a type I error rate (α) of 5% for significance in the discovery data set, based on the number of proteins or metabolites being individually tested in each analysis. The results were validated in an independent data set, again with correction for the number of tests performed.

Kaplan-Meier estimations illustrated events (deaths) from time of sampling in relation to lipoprotein levels. The log-rank test was used to compare survival distributions. Receiver operating characteristic (ROC) curves were used to assess the prognostic discriminatory ability of specific lipoproteins.

Plasma proteomic measurements were available for 173 patients in total, 122/127 in the discovery and 51/77 in the validation cohorts. Linear regression models were used to assess the relationship between levels of individual lipoprotein subclasses and individual proteins (as a continuous variable) correcting for potential confounders including age, gender, ethnicity, PAH-targeted drugs, statin therapy, oral anticoagulation, diabetes, renal and hepatic impairment, survival status and batch. Associations between lipoprotein and protein levels were also assessed using Spearman’s rank correlation. Enrichment analysis of identified proteins was conducted using the gene ontology enrichment analysis tool.^28^ Network analysis of proteins and lipoprotein particles was performed based on second order partial Spearman’s rank correlation coefficients.^29^ Networks were visualised using Cytoscape V.3.6.1.^30^

Data are presented as absolute numbers, percentages, mean with SD or median with IQR. The threshold for significance is presented separately for each multiple testing approach. P values are presented unadjusted unless stated otherwise. Column bars, Bland-Altman and forest plots were visualised using GraphPad Prism V.5 (GraphPad Software, La Jolla, California, USA). Statistical analyses were performed with IBM SPSS Statistics V.23 (IBM Corp), Microsoft Excel 2013 (Microsoft, Redmond, Washington, USA) and R-V.3.4.1 with the associated packages ‘Hmisc’ and ‘survival’ (R Foundation for Statistical Computing, Vienna, Austria).

## Results

### Characteristics of cohorts

The characteristics of the discovery and validation patient cohorts are presented in table 1. There were 43/127 (34%) and 45/77 (58%) deaths during a median follow-up time of 4.89 (IQR 3.76–5.22) and 3.4 (IQR 1.59–5.16) years, respectively.

**Table 1:** Characteristics of study subjects

	Discovery cohort	Validation cohort
	n=127	n=77
Age, years	54±17	57±17
Body mass index, kg/m^2^	28±7	29±8
Female, n (%)	89 (70)	47 (61)
Caucasian ethnicity, n (%)	111 (87)	58 (75)
Vasoresponder, n (%)	8 (6)	–
Heritable PAH, n (%)	9 (7)	2 (3)
Baseline haemodynamics		
Mean pulmonary artery pressure, mm Hg	52±16	49±12
Pulmonary artery wedge pressure, mm Hg	12±5	11±5
Pulmonary vascular resistance, WU	11±6	11±5
Cardiac output, L/min	4.3±1.6	3.9±1.6
Functional capacity		
6MWD, m	313±158	263±162
WHO-FC III/IV, n (%)	94 (74)	55 (72)
Laboratory investigations		
NT-proBNP, ng/mL	711±839	1316±1287
Bilirubin, μmol/L	14±10	18±9
Creatinine, mg/dL	86±31	99±50
C reactive protein, mg/dL	5±7	9±12
Medication		
Phosphodiesterase type 5 inhibitor, n (%)	97 (76)	42 (54)
Endothelin receptor antagonist, n (%)	51 (40)	47 (61)
Prostacyclin analogue, n (%)	23 (18)	8 (10)
Statin, n (%)	38 (30)	21 (27)
Oral anticoagulation, n (%)	100 (79)	51 (66)
ACE inhibitor, n (%)	19 (15)	23 (30)
Diuretics, n (%)	58 (46)	48 (62)
Aldosterone antagonists, n (%)	47 (37)	33 (43)
Comorbidities		
Atrial fibrillation/flutter, n (%)	21 (17)	16 (21)
Diabetes mellitus, n (%)	24 (19)	19 (25)
Coronary artery disease, n (%)	19 (15)	21 (27)
COPD, n (%)	10 (8)	16 (21)
Systemic hypertension, n (%)	38 (30)	25 (33)

Means and SDs or counts are given.

COPD, chronic obstructive pulmonary disease; 6MWD, 6 min walk distance; NT-proBNP, N-terminal pro-brain natriuretic peptide; WC, Wood units; WHO-FC, WHO functional class.

### Agreement and distribution of lipoprotein measurements in patients with PAH

NMR and clinical measurements of the principal lipoprotein classes (LDL and HDL cholesterol) showed good agreement as indicated by correlation analyses (r=0.83–0.94, all p<0.0001, online supplementary table S2, figure S1A) and assessed by the Bland-Altman method (online supplementary figure S1B). Mean plasma HDL cholesterol was 60 mg/dL (SD ±13) in the discovery and 46 mg/dL (±12) in the validation cohort, respectively. Mean plasma LDL cholesterol was 106 mg/dL (±34) and 80 mg/dL (±28), respectively.

### Identification and validation of lipoproteins associated with survival in patients with PAH

**Figure 1:**
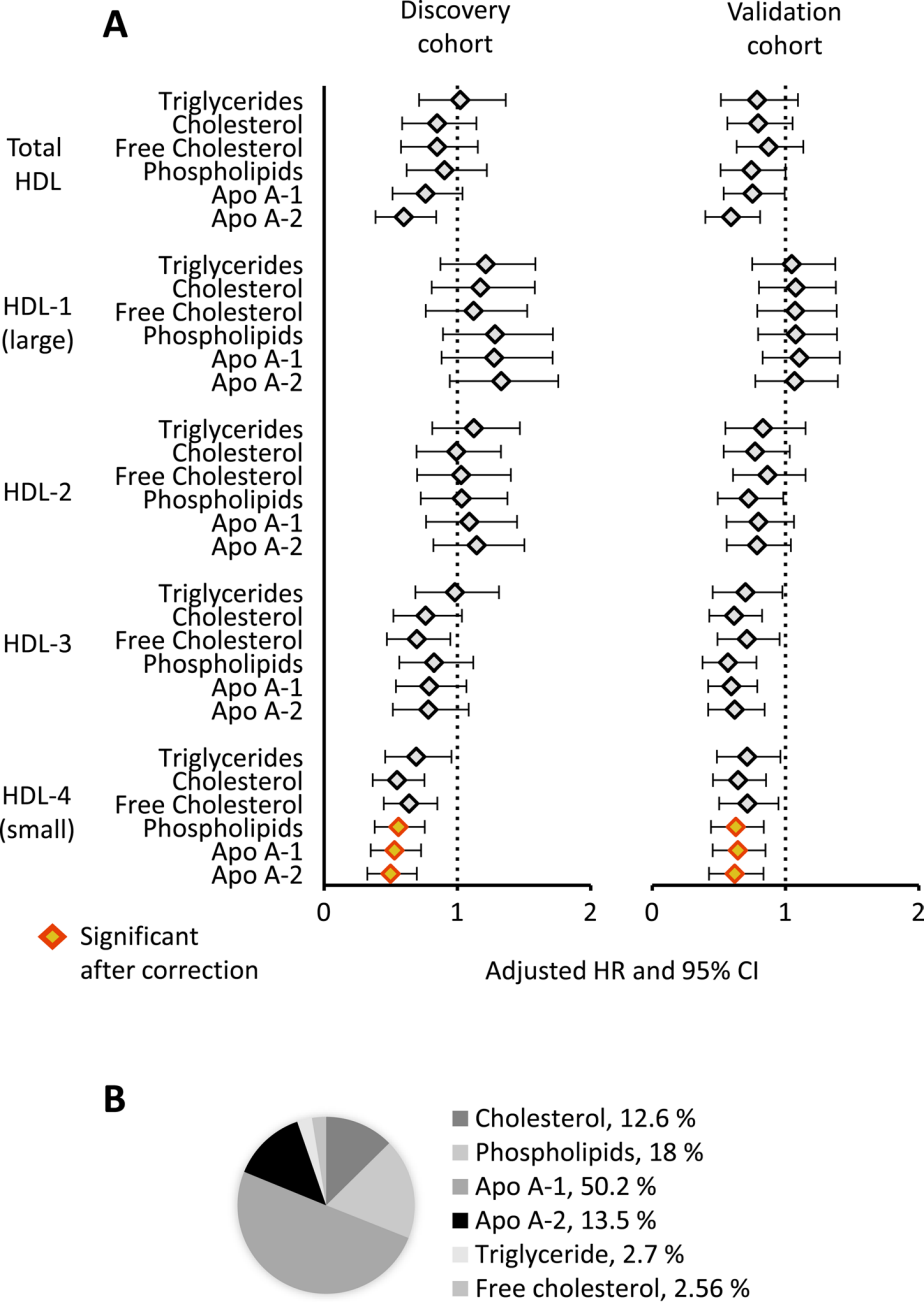
Prognostic HDL subclasses. (A) HRs and 95% CI after correcting for age, diuretic treatment and the presence of atrial fibrillation/flutter are shown for all HDL subclasses in the discovery and validation cohorts. Red/yellow indicates HDL subclasses, which meet Bonferroni correction in both cohorts. (B) Average composition of the HDL-4 subclass is shown across patients with PAH. Apo, apolipoprotein; CI, confidence interval; HDL, high-density lipoprotein; HR, hazard ratio; PAH, pulmonary arterial hypertension.

In the discovery cohort, circulating levels of 3 out of the 105 lipoproteins measured were associated with survival independent of confounding factors (all p<4.8×10^−4^, table 2 and online supplementary table S3). These lipoproteins belonged to the same lipoprotein subclass (HDL-4)—HDL-4-Apo A-1, HDL-4-Apo A-2 and HDL-4-phospholipids. None of the other lipoprotein classes or subclasses tested, including HDL cholesterol and LDL cholesterol, were prognostic (online supplementary table S3). The same three lipoproteins from the HDL-4 subclass were also associated with survival in the validation cohort, again independent of confounders (all p<1.7×10^−2^, table 2). The HDL-4 subclass is the smallest in size and highest in density in the spectrum of HDL particles. The hazard ratios (HR) for principal HDL classes and different HDL subclasses (ranging from large HDL-1 to small HDL-4) were similar in both cohorts, with higher levels of HDL-4 consistently associated with significantly better outcomes (figure 1A). HDL-4-Apo A-2 exhibited the most significant association with survival (table 2) and constitutes 13.5% of the total concentration of HDL-4 subclass lipoproteins, with HDL-4-Apo A-1 being the most abundant lipoprotein in this subclass (figure 1B).

**Table 2:** Cox proportional hazard analyses of four prognostic lipoproteins (full list of primary analyses are given in the online supplementary table S3), and secondary analyses with known prognostic factors, principal lipoprotein classes and CRP

	Discovery cohort			Validation cohort		
	HR	95% CI	P values	HR	95% CI	P values
	Primary analysis: adjusted for age, diuretic treatment and atrial fibrillation					
HDL-4-Apo A-2	0.48	0.33 to 0.7	1.35×10^–4^	0.6	0.43 to 0.84	2.67×10^–3^
HDL-4-Apo A-1	0.51	0.35 to 0.73	2.41×10^–4^	0.62	0.46 to 0.85	3.03×10^–3^
HDL-4-phospholipids	0.54	0.38 to 0.76	3.75×10^–4^	0.61	0.44 to 0.84	2.33×10^–3^
	Secondary analysis: adjusted for 6MWD and NT-proBNP					
HDL-4-Apo A-2	0.55	0.36 to 0.84	6.03×10^–3^	0.61	0.43 to 0.86	5.18×10^–3^
HDL-4-Apo A-1	0.63	0.42 to 0.96	3.29×10^–2^	0.65	0.47 to 0.92	1.38×10^–2^
HDL-4-phospholipids	0.63	0.42 to 0.94	2.38×10^–2^	0.64	0.46 to 0.89	7.91×10^–3^
	Secondary analysis: adjusted for HDL cholesterol, LDL cholesterol and CRP					
HDL-4-Apo A-2	0.57	0.39 to 0.84	4.53×10^–3^	0.54	0.34 to 0.86	9.32×10^–3^
HDL-4-Apo A-1	0.60	0.40 to 0.90	1.41×10^–2^	0.53	0.33 to 0.85	8.28×10^–3^
HDL-4-phospholipids	0.61	0.41 to 0.91	1.53×10^–2^	0.51	0.32 to 0.82	5.03×10^–3^

Apo, apolipoprotein; CI, confidence interval; CRP, C reactive protein; HDL, high-density lipoprotein; HR, hazard ratio; LDL, low-density lipoprotein; 6MWD, 6 min walk distance; NT-proBNP, N-terminal pro-brain natriuretic peptide.

We next tested the sensitivity of the HDL-4 subclass newly identified to clinical measurements of exercise capacity and inflammation. All three HDL-4 lipoproteins identified in the survival analysis (HDL-4-Apo A-1, HDL-4-Apo A-2 and HDL-4-phospholipids) were associated with outcomes independent of 6MWD and NT-proBNP level, in both the discovery and validation cohorts (all p<0.05, table 2). These three lipoproteins were also prognostic independent of the principal lipoprotein classes, HDL cholesterol and LDL cholesterol, and of the inflammatory marker, CRP, in both cohorts (all p<0.05, table 2). This emphasises the value of the lipoprotein subclasses measured by NMR above the principal classes usually captured in clinical testing.

**Figure 2:**
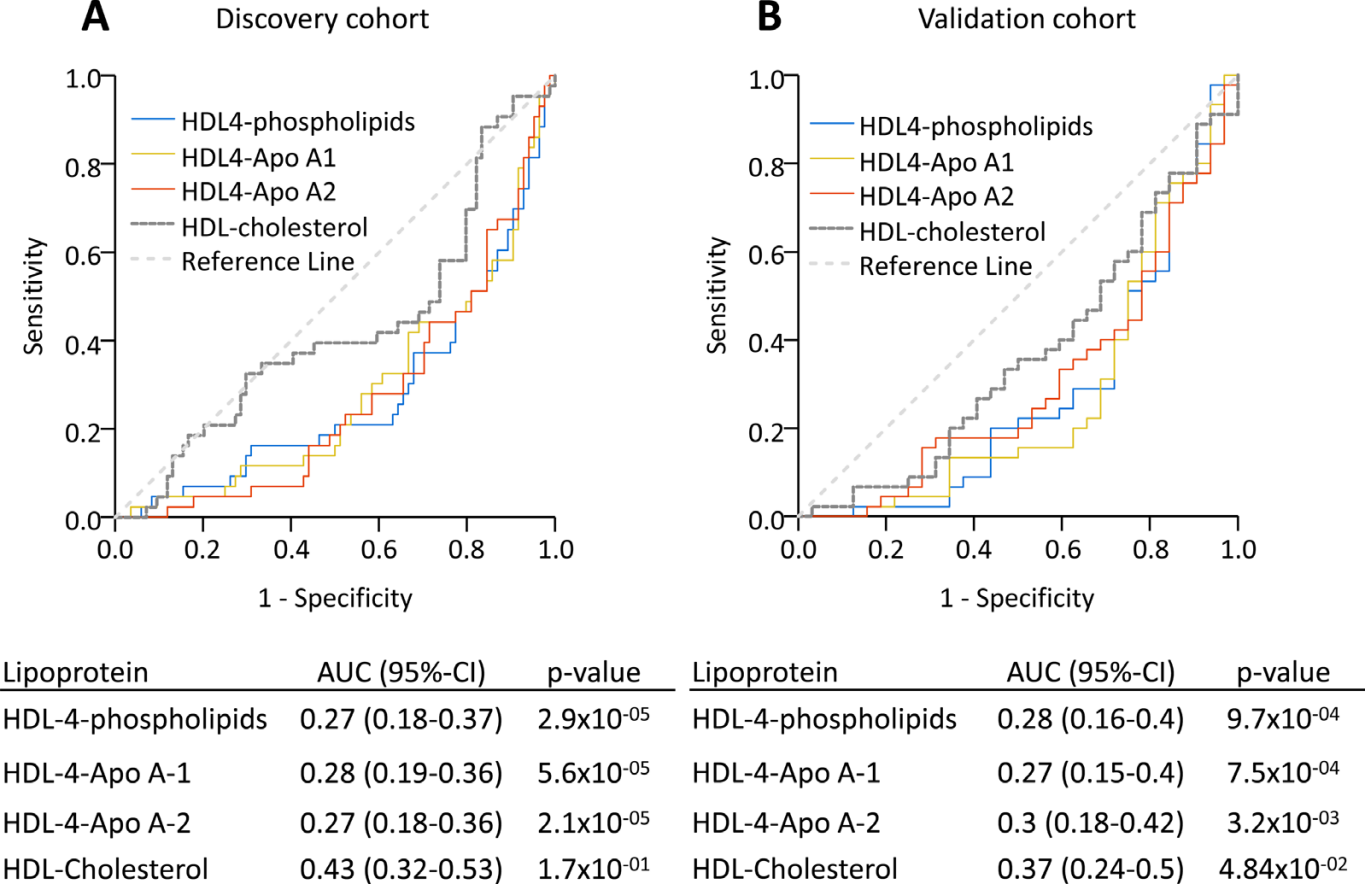
HDL-4 lipoproteins identified non-survivors in PAH. ROC assessing the ability to discriminate survivors and non-survivors for HDL-4-phospholipids, HDL-4-Apo A-1 and HDL-4-Apo A-2, as well as overall HDL cholesterol are shown in the discovery and validation cohorts. Apo, apolipoprotein; AUC, area under the curve; HDL, high-density lipoprotein; PAH, pulmonary arterial hypertension; ROC, receiver operating characteristic.

To determine the ability of the lipoprotein subclasses to distinguish survivors and non-survivors, we performed ROC analyses. The three HDL-4 lipoproteins showed similar performance in the discovery and validation cohorts (area under the curve 0.27–0.30, figure 2), while total HDL cholesterol was not discriminative in either cohort (area under the curve 0.43–0.37, figure 2).

### Analysis of potential clinical confounders

We investigated the importance of lipid-lowering treatments on our analyses. Patients treated with statins at the time of lipoprotein measurement had lower levels of LDL subclasses but not HDL subclasses (including HDL-4), and statin treatment was not a predictor of outcomes in either cohort (online supplementary figure S2, table S1). This suggests little impact of statins on our main findings.

**Figure 3:**
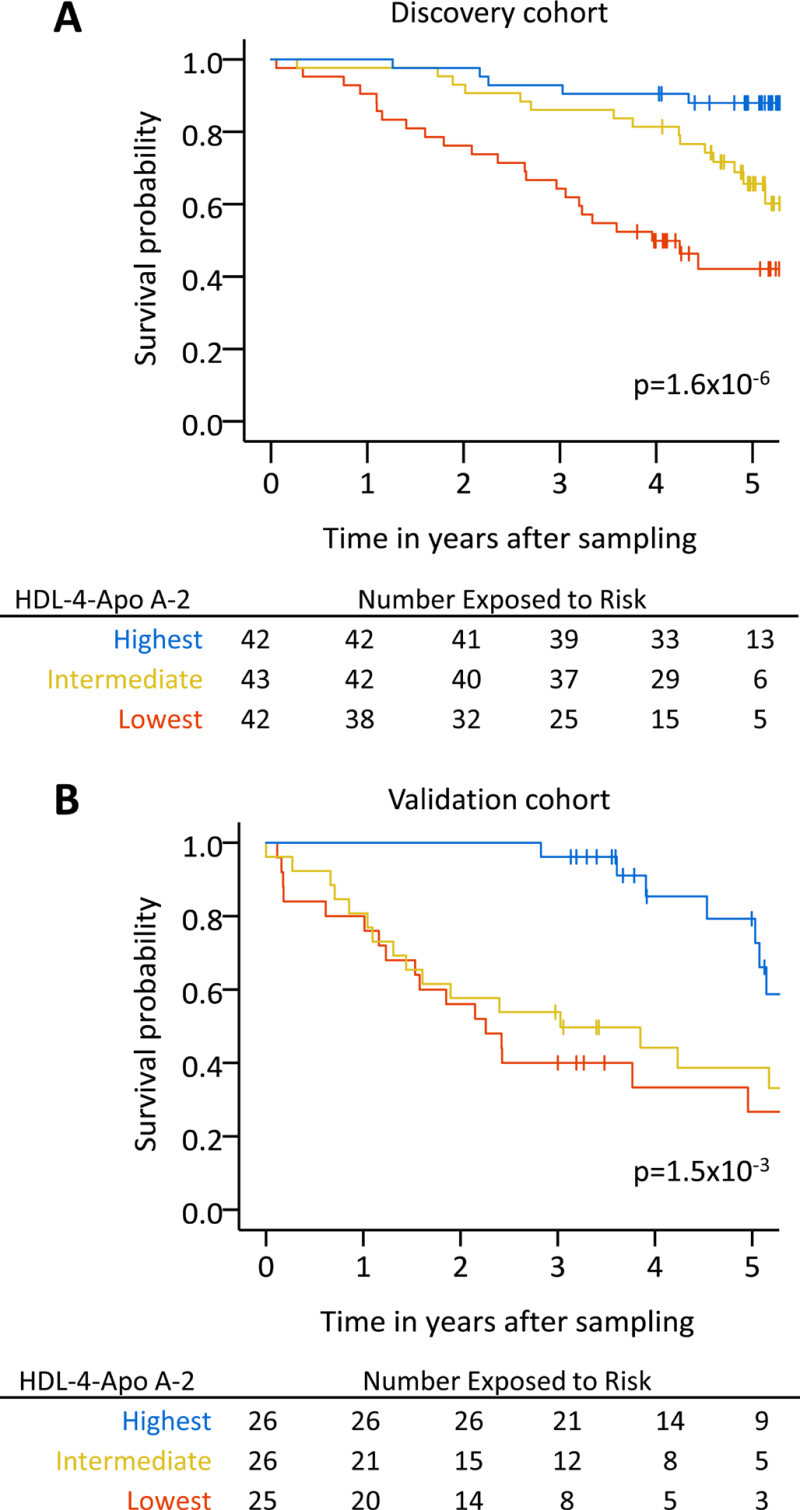
High, intermediate and low levels of HDL-4-Apo A-2 are prognostic in PAH. Kaplan-Meier survival estimates for patients with PAH separated into tertiles based on high (blue, range 22–33 mg/dL), intermediate (yellow, range 18–22 mg/dL) and low (orange, range 7–18 mg/dL) HDL4-Apo A-2 levels are shown in the discovery and validation cohorts. Apo, apolipoprotein; HDL, high-density lipoprotein; PAH, pulmonary arterial hypertension.

Kaplan-Meier survival estimates for patients with PAH separated into HDL-4-Apo A-2 tertiles significantly stratified outcomes in both cohorts (p<0.05, figure 3). We also assessed the prognostic value of HDL-4-Apo A-2 in a subgroup of patients with PAH and limited cardiovascular comorbidities (less than three risk factors for left heart failure).^22^ HDL-4–Apo A-2 remained strongly prognostic in both cohorts (p<0.05, online supplementary figure S3). Similarly with incident patients sampled around the time of diagnosis, reduced levels of HDL-4-Apo A-2 tended to be associated with poor outcomes (online supplementary figure S4).

### Defining small HDL and proteome associations in PAH

**Figure 4:**
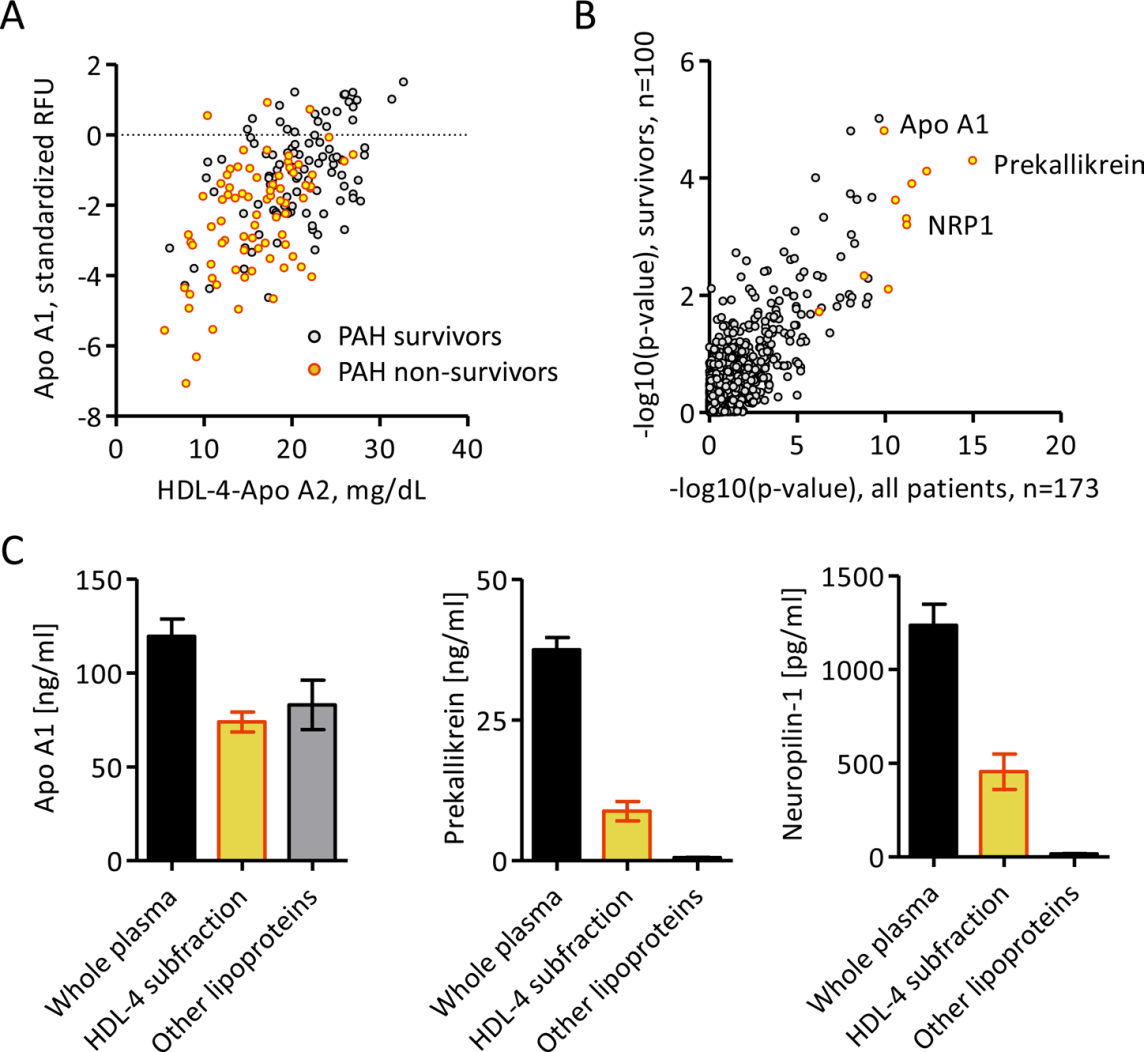
Proteins related to HDL-4-Apo A-2. (A) Scatter plot showing the relation of Apo A-1 and HDL-4-Apo A-2 in the combined discovery and validation cohort, in which non-survivors are marked in yellow/red, with statistical result from Spearman’s rank correlation. (B) Scatter plot showing the significance from Spearman’s rank correlation between HDL-4-Apo A-2 levels and proteomic measurements in all PAH patients and PAH survivors, in the combined discovery and validation cohort. Proteins marked in yellow/red significantly related with HDL4-Apo A-2 after Bonferroni correction, independent of confounding factors in linear regression models. (C) Bar plots (mean±SEM) showing the presence of Apo A-1, prekallikrein and NRP1 in whole plasma and lipoprotein fractions obtained by ultracentrifugation corresponding to small and dense HDL-4 from eight patients with PAH. Apo, apolipoprotein; HDL, high-density lipoprotein; NRP1, neuropilin-1; PAH, pulmonary arterial hypertension; RFU, relative fluorescence unit; SEM, SE mean.

To explore the contribution of the HDL proteome in PAH, HDL-4-Apo A-2 levels, which exhibited the most significant association with survival, were first related to measurements of 1124 circulating proteins. Nine proteins were found to associate with HDL-4-Apo A-2 (all p<4.5×10^−5^ independent of confounders in linear regression models), including the Apo A-1 protein itself (figure 4A, table 3 and online supplementary table S4). Association of these proteins with HDL-4-Apo A-2 was similar in survivors and non-survivors (figure 4B). No additional protein was found using levels of the other two HDL-4 subclasses, HDL-4-Apo A-1 or HDL-4-phospholipids (online supplementary figure S5).

**Table 3:** Linear regression models of nine proteins associated with the HDL-4-Apo A-2 particle adjusted for possible confounding factors including demographics, treatments, comorbidities and survival status

Proteins	Adjusted linear regression models against HDL-4-Apo A-2		
	Beta	SE	P values
Prekallikrein	0.59	0.10	4.86×10^–8^
Apo A-1	0.68	0.12	2.08×10^–7^
Alpha-2-antiplasmin	0.51	0.09	3.39×10^–7^
CNDP1	0.69	0.13	4.58×10^–7^
Coagulation factor XI	0.69	0.13	6.12×10^–7^
Afamin	0.59	0.13	1.7×10^–5^
Neuropilin-1	−0.50	0.11	1.8×10^–5^
Growth hormone receptor	0.43	0.09	1.37×10^–5^
Alpha-2-HS-glycoprotein	0.42	0.09	2.04×10^–5^

Apo, apolipoprotein; CNDP1, carnosine dipeptidase 1; HDL, high-density lipoprotein.

Five of the nine proteins, namely, Apo A-1, alpha-2-antiplasmin, coagulation factor XI, afamin and alpha-2-HS-glycoprotein, have previously been identified in HDL subfractions.^18 20 31^ Prekallikrein levels showed the highest association with HDL-4-Apo A-2 (p=4.9×10^−8^, table 3 and online supplementary table S4) and were statistically associated with each of the three HDL-4 subclasses in linear regression models (HDL-4-Apo A-1, HDL-4-Apo A-2 and HDL-4-phospholipids, online supplementary figure S4). Newly identified neuropilin-1, which showed the only significant inverse relation, was only detected with HDL-4-Apo A-2. Using ultracentrifugation to separate the plasma subfraction corresponding to small HDL-4, we were able to confirm the physical presence of prekallikrein and neuropilin-1 with Apo A-1 in this subfraction (figure 4C).

**Figure 5:**
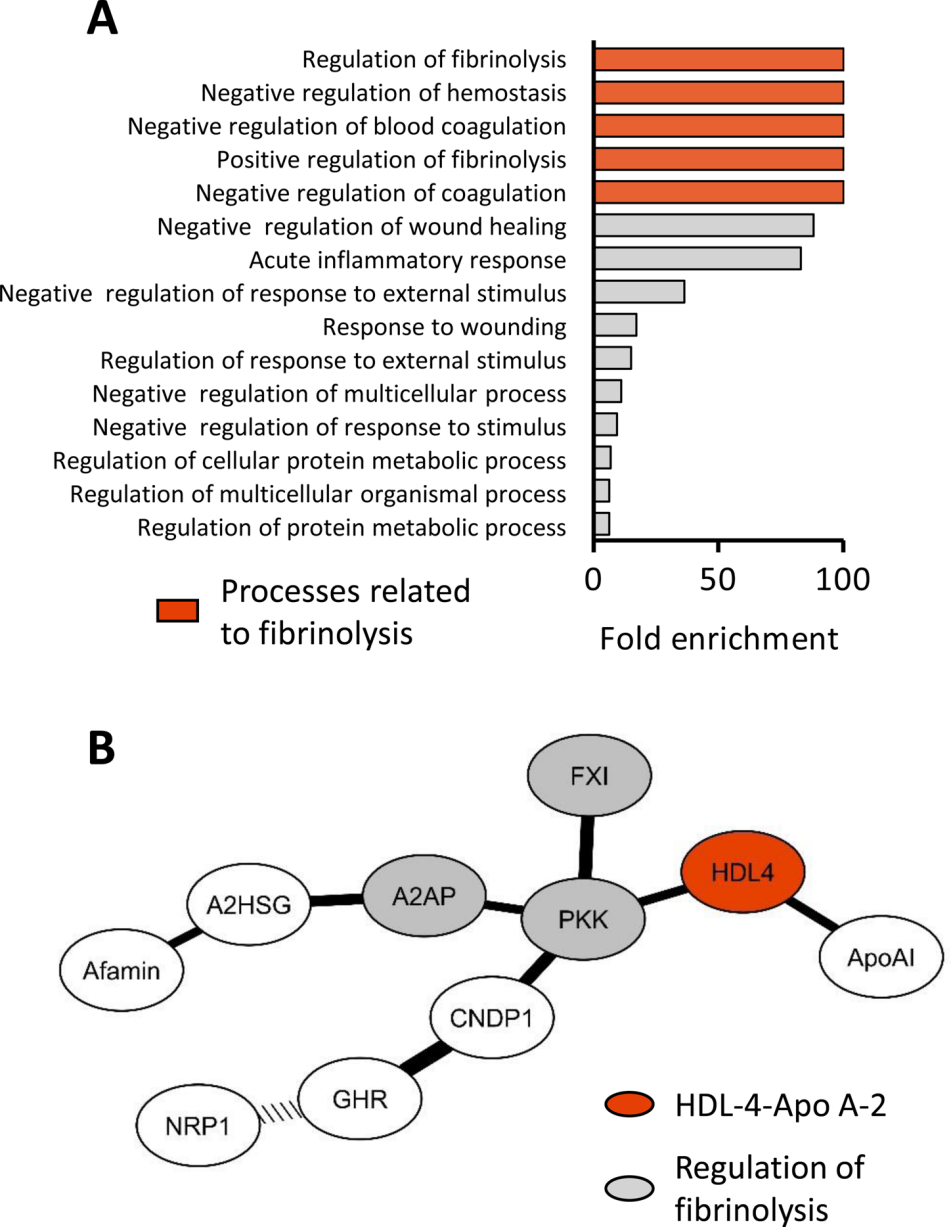
Enrichment and network analyses of proteins linked to small HDL-4-Apo A-2. (A) Significantly enriched biological processes related to the nine proteins associated independently with HDL-4-Apo A-2 in linear regression models. Pathways are in order of the corresponding fold enrichment. Highlighted in red are those biological process related to fibrinolysis. (B) Network analysis of nine proteins identified based on second order partial Spearman’s rank correlation coefficients (all p<0.0001). Highlighted in grey are proteins, which function is related to regulation of fibrinolysis, and in red is HDL-4-Apo A-2. The width of connection lines is graded according to the strength of correlation. The dotted connection line indicates an inverse relation. A2AP, alpha-2-antiplasmin; Apo, apolipoprotein; A2SG, alpha-2-HS-glycoprotein; CNDP1, carnosine dipeptidase 1; FXI, factor XI; GHR, growth hormone receptor; HDL, high-density lipoprotein; NRP1, neuropilin-1; PKK, prekallikrein.

Functional annotation of the nine proteins associated with the small HDL-4-Apo A-2 revealed an over-representation of proteins involved in the regulation of fibrinolysis, including prekallikrein, coagulation factor XI and alpha-2-antiplasmin (all corrected p<0.05, figure 5A, online supplementary table S5). When restricting the analysis to proteins previously reported or physically confirmed (7/9), the functional annotation displayed identical results (data not shown).

To determine which of the nine proteins were most closely associated with HDL-4-Apo A-2 and each other, we performed a network analysis based on partial correlation coefficients. Prekallikrein formed a central hub connecting small HDL-4 particle to factor XI and alpha-2-antiplasmin (all p<0.0001, figure 5B).

## Discussion

This study provides a comprehensive analysis of plasma lipoprotein subclasses in PAH. To our knowledge, it is the first to use a metabolomics approach to explore lipoprotein profiles in this disorder and to identify and validate the association of a small and highly dense HDL subclass with clinical outcomes. Levels of the small HDL-4 subclass, with Apo A-1, Apo A-2 and phospholipids as the main components, were identified as robust prognostic factors in PAH.

The clinical significance of altered plasma lipoprotein levels in pulmonary hypertension has been unclear and studies of the relationship between circulating HDL cholesterol levels and survival in patients with PAH have produced contrasting conclusions.^11–14^ Our data suggest that HDL subclasses differ in their contribution to the pathology of PAH; only plasma small HDL-4 levels were independently prognostic in patients with PAH. HDL-4 incorporates the previously described HDL3b and HDL3c subclasses.^8 15^ These have been implicated in several biological pathways relevant to PAH, including upregulation of endothelial nitric oxide synthase promoting vasodilatation,^32^ anti-inflammatory effects through decreased expression of endothelial adhesion molecules, decreased oxidation of LDL, antiapoptotic effects on the endothelium and activation of fibrinolysis.^33^

Increasing evidence implicates the protein content of HDL particle as functionally important in cardiovascular and respiratory disease.^8 34^ Mass spectrometry-based proteomic analysis has revealed an enrichment of acute-phase response proteins in the small HDL subclass in patients with coronary artery disease,^18^ and demonstrated that the protein content influences oxidation.^20^ The Apo A-1 content of HDL has both antioxidant and antiapoptotic effects in endothelial cells.^35^ We found that nine plasma proteins, including Apo A-1, statistically associate with the small Apo A-2-rich HDL-4 subclass. Only five out of nine identified proteins (alpha-2-antiplasmin, alpha-2-HS-glycoprotein, afamin, coagulation factor XI and Apo A-1) have previously been reported to be bound to HDL.^18 20^ Several of the proteins identified (prekallikrein, factor XI and alpha-2-antiplasmin) regulate fibrinolysis, a function previously attributed to the small HDL subclass.^36^ Prekallikrein, the physical presence of which was confirmed in the isolated HDL-4 subfraction, formed a central hub in a network generated from the nine proteins identified and HDL-4. Prekallikrein can be cleaved by activated factor XII to form plasma kallikrein, which subsequently digests the non-enzymatic cofactor high-molecular-weight kininogen to release bradykinin, the end product of the kallikrein–kinin pathway.^37^ Both prekallikrein and bradykinin are involved in fibrinolysis, vascular inflammation and the regulation of vascular tone.^37^

Recently the promoter of the gene encoding a major cellular lipid transporter, ABCA1, was shown to be hypermethylated in PAH, resulting in the downregulation of ABCA1 messenger RNA and protein in pulmonary arterial endothelial cells from patients with idiopathic PAH.^38^ This protein accelerates the transfer of lipids to Apo A-1-containing lipoproteins, the rate-limiting step in the generation of large lipid-rich HDL particles, and a decrease in its expression may represent a protective mechanism to enrich beneficial small HDL particles in PAH.

Therapeutic strategies designed to increase HDL cholesterol levels include the use of niacin and fibrates, which also induce the transcription of Apo A-1.^39^ Direct inhibition of cholesteryl ester transfer protein has been proposed as a means of specifically increasing HDL cholesterol in cardiovascular disease, but clinical trials of inhibitors such as torcetrapib or evacetrapib have shown only limited improvements in cardiovascular outcomes.^40 41^ Looking more specifically at the HDL subclasses, some studies have reported that the use of atorvastatin significantly increases large HDL, but not medium or small HDL subclasses.^42^ Lipoproteins such as Apo A-1 represent an alternative potential therapeutic target, with Apo A-1-mimetics preventing the development of experimental pulmonary hypertension,^9 10^ and their use is currently being assessed in lung diseases such as asthma and emphysema.^43^ Our study raises the possibility that the potential therapeutic benefit of Apo A-1 manipulation may extend beyond lung disease to the pulmonary vasculature and so would be of interest to study in the context of pulmonary hypertension.

### Limitations

The majority of patients in this study were on treatment, that is, had prevalent disease, however, we observed similar trends in a survival analysis of incident patients recruited close to the time of diagnosis. Corrections were made for the potential treatment effects of PAH-targeted drugs, oral anticoagulation and statin therapy, as well as demographics and comorbidities. The patients were sampled in a non-fasting state, as supported by recent guidelines recommending that non-fasting samples can be used for plasma lipid analysis.^44^ Proteins associated with the HDL-4 subfraction may also circulate free in the plasma.

## Summary and conclusions

Reduced plasma levels of small Apo A-2-rich HDL-4 are independently linked with higher mortality in PAH, supporting a biologically important role in the natural history of this disorder. Apolipoproteins and other HDL-4-associated proteins, including prekallikrein and alpha-2-antiplasmin, regulate biological mechanisms relevant to PAH, such as fibrinolysis and vasodilation. Circulating small HDL serves as vehicle for these proteins. Our results support further investigation into the potential therapeutic benefit of increasing the levels of small HDL and its associated proteins in PAH.
