# Supplementary material for: Reduced plasma levels of small HDL particles transporting fibrinolytic proteins in pulmonary arterial hypertension
Source: Thorax. 2018 Nov 26;74(4):380–9. doi: 10.1136/thoraxjnl-2018-212144 (PMC6475111; doi:10.1136/thoraxjnl-2018-212144)
Supplement: Supplementary file 1 [file thoraxjnl-2018-212144supp001.pdf]

Online Data Supplement

Reduced Plasma Levels of Small HDL Particles Transporting Fibrinolytic Proteins In Pulmonary Arterial Hypertension

Lars Harbaum, Pavandeep Ghataorhe, John Wharton, Beatriz Jiménez, Luke S. G. Howard, J. Simon R. Gibbs, Jeremy K. Nicholson, Christopher J. Rhodes and Martin R. Wilkins

Content of data supplement

Supplement Methods – page 1-2

Figure S1 – page 3

Figure S2 – page 4

Figure S3 – page 5

Figure S4 – page 6

Figure S5 – page 7

Table S1 – page 8

Table S2 – page 9

Table S3 – page 10-12

Table S4 – page 13-38

Table S5 – page 39

Supplement Methods and Marterial

Nuclear magnetic resonance (NMR) spectroscopy

Plasma samples were thawed, vortexed, centrifuged at 16,000g for 5 minutes at 4^0C and distributed in Riplate™ 96-well 1ml plates (Ritter, GmbH, Schwabmünchen, Germany). Aliquots of plasma (300 µl per well) were then mixed 1:1 with 300 µL phosphate buffer (pH 7.4), containing the reference standard trimethylsilylpropionic acid, in flow-injection plates. (1). A representative quality control sample was included in all plates at regular intervals. NMR data was acquired at 310K, using a 600 MHz Avance III NMR Spectrometer equipped with a SampleJet sample handler, which stores samples at 4^0C high degree shimming coil and z-gradients probes (BrukerBioSpin). Data was acquired with a standard one-dimensional solvent suppression pulse sequence including two suppression delays, the Carr–Purcell-Meiboom–Gill (CPMG) pulse sequence and a two dimensional *J*-resolved (*J*-res) experiment [1]. Spectral width was set to 30 ppm with the central of the window in the water signal. The relaxation delay was 4 s and 32 scans were acquired for the monodimensional experiments while 2 scans were acquired for the *J*-res. Each of the experiments was optimised to last 4 mins.

The training sample set, which was used to construct the linear regression models for the Bruker IVDr Lipoprotein Subclass Analysis (B.I.-LISA™), applied the following densities to separate lipoproteins subclasses by ultracentrifugation [2]. The densities of the principle lipoprotein classes were: VLDL (<1.006 kg/l), IDL (1.006-1.019 kg/l), LDL (1.019-1.063 kg/l) and HDL (1.063-1.210 kg/l). The densities of the lipoprotein subclasses were: LDL-1 (<1.031 kg/l), LDL-2 (1.031-1.034 kg/l), LDL-3 (1.034-1.037 kg/l), LDL-4 (1.037-1.040 kg/l), LDL-5 (1.040-1.044 kg/l), LDL-6 (>1.044 kg/l), HDL-1 (1.063-1.100 kg/l), HDL-2 (1.100-1.112 kg/l) HDL-3 (1.112-1.125 kg/l), HDL-4 (1.125-1.210 kg/l). VLDL-1 (top) to VLDL-6 (bottom) had values based on flotation constants (Sf) ranging from 400 to 20. For each of the lipoprotein subclasses, the lipid content of cholesterol (Ch), free cholesterol (FC), phospholipids (PL), Apo A1, Apo A2 and triglycerides (TG)) was analysed in the training set and introduced into the regression.

Supplement references

1. Beckonert O, Keun HC, Ebbels TM, et al. Metabolic profiling, metabolomic and metabonomic procedures for NMR spectroscopy of urine, plasma, serum and tissue extracts. *Nature protocols* 2007;2(11):2692-703. doi: 10.1038/nprot.2007.376 [published Online First: 2007/11/17]
2. Mihaleva VV, van Schalkwijk DB, de Graaf AA, et al. A systematic approach to obtain validated partial least square models for predicting lipoprotein subclasses from serum NMR spectra. *Analytical chemistry* 2014;86(1):543-50. doi: 10.1021/ac402571z [published Online First: 2013/12/11]

Figure S1

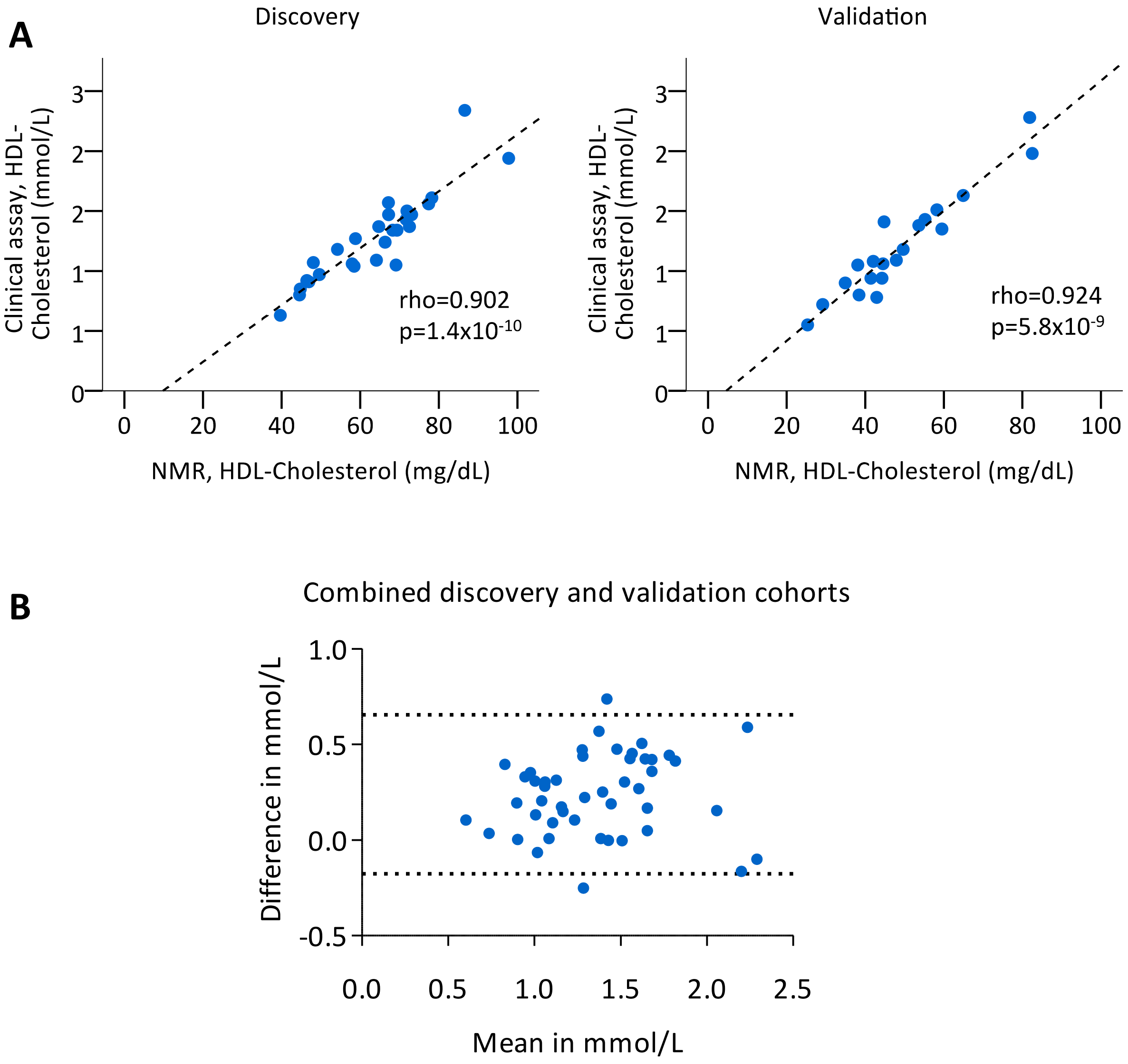

Figure S1 – Nuclear magnetic resonance (NMR) spectroscopy measurements of HDL cholesterol levels correlate to clinical precipitation assay. (A) Scatter plot of HDL cholesterol measured from NMR lipoprotein analysis and clinical assay measurements are shown in the discovery (n= 27) and validation cohorts (n=20). Statistics shown are from Spearman's Rank test. (B) Bland-Altman plot comparing HDL cholesterol measurements from clinical precipitation assay and NMR in the combined discovery and validation cohorts (n=47). 95% confidence interval is shown in dotted horizontal lines. Units were converted from mg/dL to mmol/L using the factor 38.67 (e.g. 1 mmol/L = 38.67 mg/dL). High-density lipoprotein (HDL).

Figure S2

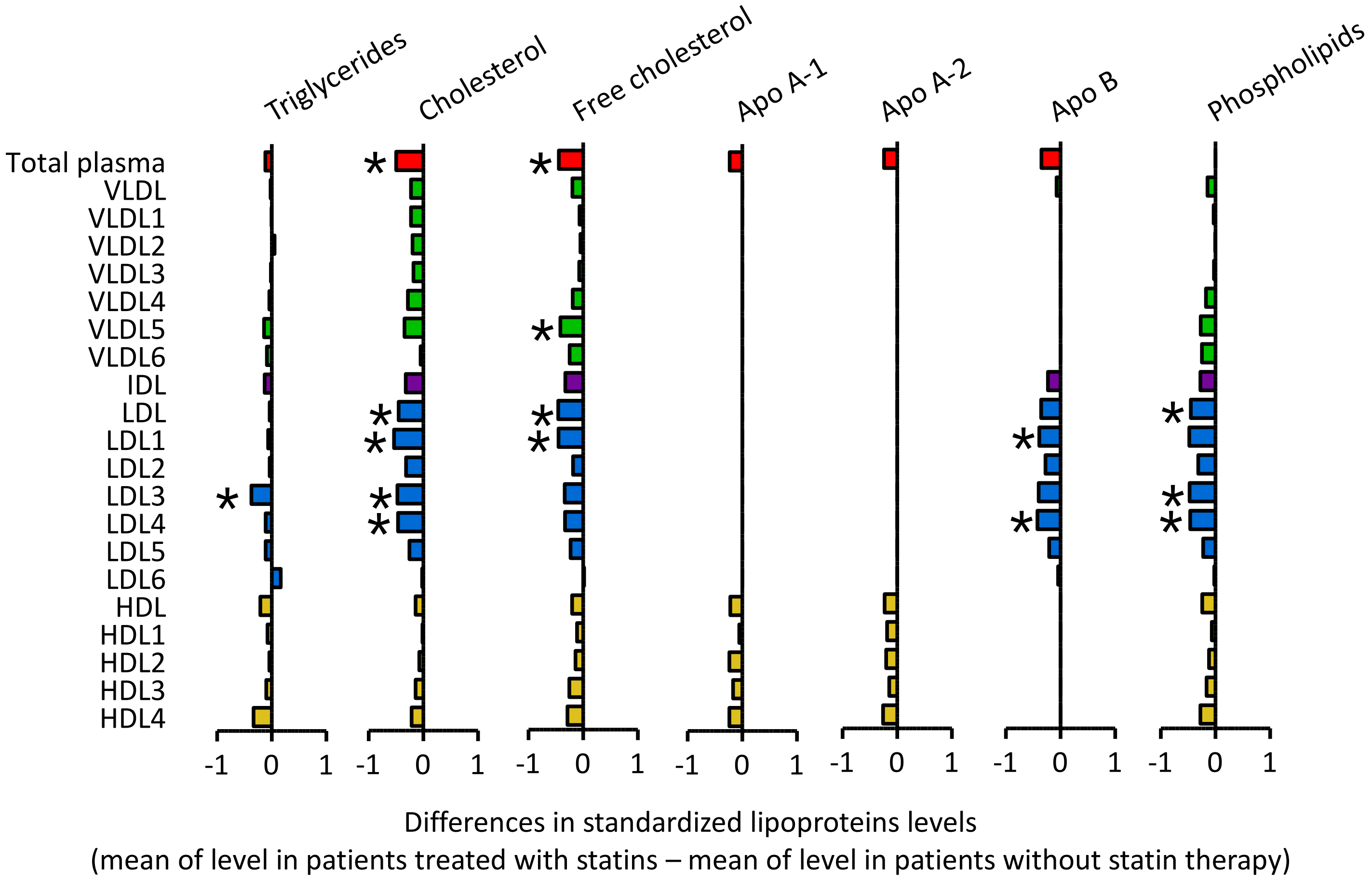

Figure S2 – Effect of statin therapy on lipoprotein profile. Differences in standardized lipoprotein levels between PAH patients treated with statins at time of blood sample (n=30) and patients without statin therapy (n=101) are shown in the discovery cohort. Lower mean difference indicates a decrease in patients on statin treatment. * indicates lipid features that are significantly different between PAH patients on and off statin therapy by Mann Whitney U test ($p<0.05$). Very low-density lipoprotein (VLDL), low-density lipoprotein (LDL), intermediate-density lipoprotein (IDL), high-density lipoprotein (HDL), apolipoprotein (Apo).

Figure S3

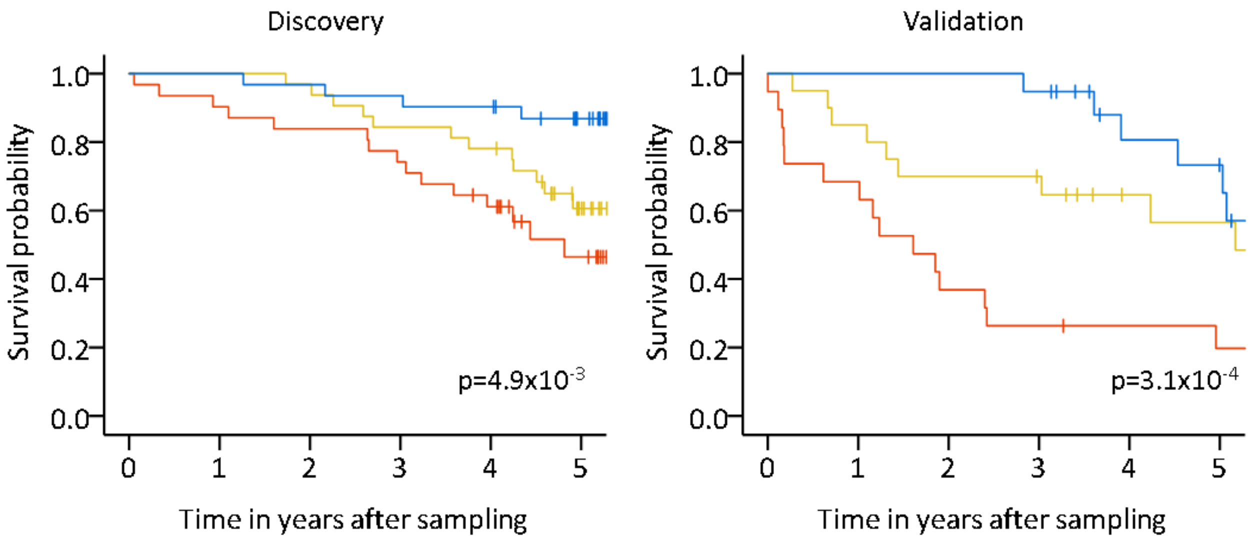

HDL-4-Apo A-2	Number Exposed to Risk					
Highest	31	31	30	29	24	10
Intermediate	32	32	31	27	21	6
Lowest	31	28	26	23	15	4

HDL-4-Apo A-2	Number Exposed to Risk					
Highest	19	19	19	16	11	6
Intermediate	20	17	14	11	8	5
Lowest	19	13	7	5	4	3

Figure S3 - High, intermediate and low levels of HDL-4-Apo A2 are prognostic in patients with PAH and limited cardiovascular risk factors. Kaplan Meier survival estimates for PAH patients separated into tertiles based on high (blue), intermediate (yellow) and low (orange) HDL-4-Apo A-2 levels are shown in the discovery (n=96) and validation cohorts (n=59) for patients with PAH and limited cardiovascular risk factors, which was defined as patients with less than three risk factors for left heart disease. High-density lipoprotein (HDL), apolipoprotein (Apo).

Figure S4

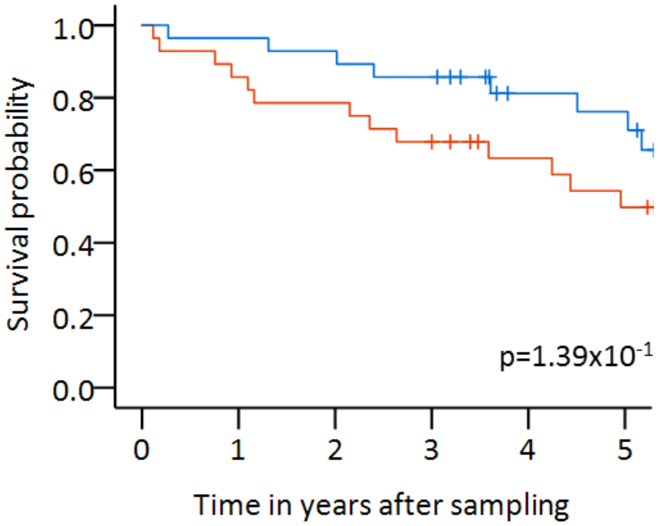

HDL-4-Apo A-2	Number Exposed to Risk					
Above median	28	27	26	21	16	9
Below median	28	24	22	17	14	6

Figure S4 - Levels of HDL-4-Apo A2 in incident cases with PAH are by trend associated with survival. Kaplan Meier survival estimates for incident PAH patients (n=56) separated by median of HDL-4-Apo A2. Samples were taken around the period of diagnosis (180 days). High-density lipoprotein (HDL), apolipoprotein (Apo).

Figure S5

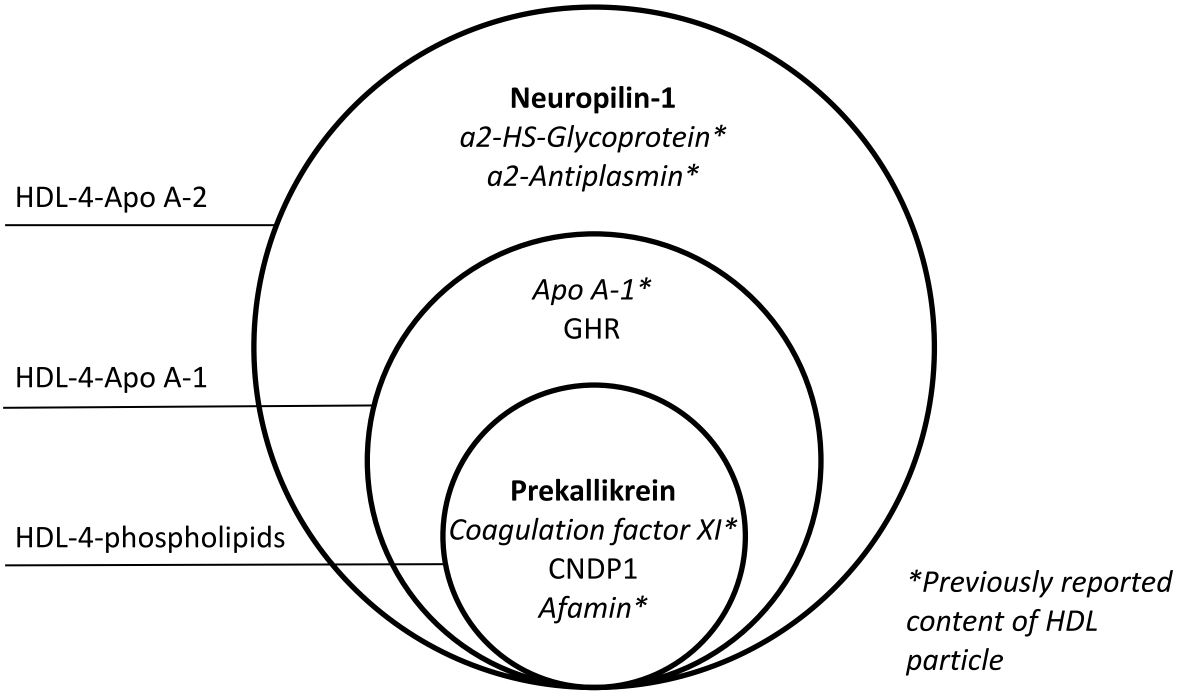

Figure S5 – Proteins identified that associate with levels of three HDL-4 subclasses in patients with PAH. Level of 1,124 circulating proteins were related to either of three HDL-4 subclasses using linear regression models in 173 patients with idiopathic or hereditable pulmonary arterial hypertension (PAH). Potential confounding factor were included as covariats, including age, gender, ethnicity, PAH-targeted drugs, statin therapy, oral anticoagulation, diabetes, renal and hepatic impairment, survival status and batch. Bonferroni correction for multiple testing was applied. Marked with asterisks are those protenis, which previously have been reported as content of HDL particles (references in the manuscript). Highlithed in bold are those proteins, which physical presence in small HDL particles were newly identified. The number of proteins identified increased from HDL-4-phopholipids to HDL-4-Apo A-2 particles. Neuroplin-1, a2-HS-glycoprotein and a2-antiplasmin were only identified with HDL-4-Apo A-2 as covariate in the linear regression model, while prekallikrein, cogulation factor XI, CNDP1 and afamin were identified with all three HDL-4 particles. High-density lipoprotein (HDL), apolipoprotein (Apo), growth hormone receptor (GHR), carnosine dipeptidase (CNDP1).

Table S1 – Cox regression analyses for all included confounding factors in the discovery and validation cohort. Marked in grey the top 3 variables, which were included in the subsequent survival analysis of lipoprotein subclasses in the discovery and validation cohort. Body mass index (BMI), chronic obstructive pulmonary disease (COPD), endothelin receptor antagonist (ERA), phosphodiesterase type 5 (PDE5).

Possible confounding factors	Discovery cohort		Validation cohort	
	Univariate Cox proportional hazard model			
	Hazard ratio	Sig.	Hazard ratio	Sig.
Atrial fibrillation/flutter	3.73 (1.96-7.09)	5.97E-05	2.24 (1.18-4.25)	1.39E-02
Age per year	1.04 (1.02-1.06)	6.10E-05	1.02 (1.00-1.04)	1.31E-02
Diuretic treatment	3.90 (2.00-7.59)	6.15E-05	2.32 (1.21-4.47)	1.17E-02
Creatinine >75 µmol/L	2.91 (1.50-5.64)	1.55E-03	3.95 (1.40-11.16)	9.49E-03
Statin	2.41 (1.33-4.36)	3.60E-03	0.93 (0.47-1.84)	8.40E-01
Systemic hypertension	2.45 (1.33-4.53)	4.16E-03	1.18 (0.62-2.24)	6.17E-01
Bilirubin >21 µmol/L	2.14 (1.10-4.16)	2.59E-02	1.96 (1.00-3.83)	5.04E-02
Female gender	0.51 (0.28-0.94)	3.14E-02	0.65 (0.37-1.17)	1.56E-01
Diabetes mellitus	1.75 (0.89-3.44)	1.02E-01	1.23 (0.63-2.38)	5.47E-01
Aldosterone antagonists	1.60 (0.89-2.90)	1.19E-01	2.06 (1.15-3.70)	1.58E-02
Oral anticoagulation	0.67 (0.35-1.31)	2.44E-01	0.94 (0.50-1.76)	8.36E-01
BMI. kg/m2	0.97 (0.93-1.02)	2.61E-01	0.98 (0.94-1.02)	2.92E-01
ERA	1.25 (0.69-2.26)	4.55E-01	0.95 (0.52-1.72)	8.57E-01
Coronary artery disease	1.26 (0.58-2.73)	5.64E-01	1.84 (0.97-3.49)	6.11E-02
COPD	1.13 (0.44-2.91)	7.80E-01	0.79 (0.38-1.65)	5.40E-01
PDE5-Inhibitor	0.95 (0.47-1.93)	8.92E-01	0.95 (0.53-1.70)	8.62E-01

Table S2 – Spearman's rank correlation matrix of lipoprotein levels from nuclear magnetic resonance spectroscopy and clinical precipitation assays. High density lipoprotein (HDL), low density lipoprotein (LDL).

**Variables**		**Discovery cohort**	**Validation cohort**
	**Correlation between clinical and NMR measures**		
Cholesterol (mmol/L)	Spearman rho	0.924	0.887
	p-value	6.10E-12	5.76E-14
	n	27	39
Triglycerides (mmol/L)	Spearman rho	0.913	0.832
	p-value	3.24E-11	5.74E-10
	n	27	35
HDL Cholesterol (mmol/L)	Spearman rho	0.902	0.924
	p-value	1.40E-10	5.80E-09
	n	27	20
LDL Cholesterol (mmol/L)	Spearman rho	0.904	0.840
	p-value	1.03E-10	3.56E-06
	n	27	20

Table S3 – Cox regression analyses for all nuclear magnetic resonance (NMR) spectroscopy based lipoprotein levels in the discovery and validation cohort adjusting for age, diuretic use and atrial fibrillation/flutter. Marked in grey are the 4 lipoprotein subclasses, which met Bonferroni correction in the discovery ($p<4.8 \times 10^{-4}$) and validation cohorts ($p<1.3 \times 10^{-2}$). High-density lipoprotein (HDL), intermediate-density lipoprotein (IDL), low-density lipoprotein (LDL), very low-density lipoprotein (VLDL), apolipoprotein (Apo), confidence interval (CI).

**Lipoprotein**	**Discovery cohort**		**Validation cohort**	
	**Multivariate Cox proportional hazard model adjusting for age. diuretic use and atrial fibrillation**			
	Hazard ratio (95%-CI)	Sig.	Hazard ratio (95%-CI)	Sig.
HDL4 - Apo A-2	0.48 (0.33-0.7)	1.35E-04	0.6 (0.43-0.84)	2.67E-03
HDL4 - Apo A-1	0.51 (0.35-0.73)	2.41E-04	0.62 (0.46-0.85)	3.03E-03
HDL4 - phospholipids	0.54 (0.38-0.76)	3.75E-04	0.61 (0.44-0.84)	2.33E-03
HDL4 - cholesterol	0.53 (0.37-0.75)	4.87E-04	0.62 (0.46-0.85)	3.28E-03
HDL4 - free cholesterol	0.62 (0.45-0.85)	3.14E-03	0.69 (0.5-0.95)	2.17E-02
Total plasma - cholesterol	0.6 (0.42-0.85)	4.03E-03	0.64 (0.46-0.9)	1.12E-02
HDL - Apo A-2	0.57 (0.39-0.84)	4.74E-03	0.57 (0.4-0.81)	1.81E-03
Total plasma - Apo A-2	0.58 (0.39-0.85)	5.04E-03	0.58 (0.41-0.83)	2.53E-03
Total plasma - free cholesterol	0.65 (0.46-0.91)	1.24E-02	0.69 (0.5-0.97)	3.11E-02
VLDL6 - phospholipids	0.6 (0.4-0.9)	1.36E-02	0.56 (0.38-0.83)	3.36E-03
VLDL6 - cholesterol	1.58 (1.1-2.27)	1.36E-02	1.16 (0.87-1.54)	3.06E-01
LDL6 - Apo B	0.65 (0.45-0.93)	1.93E-02	0.86 (0.63-1.17)	3.28E-01
Total plasma - Apo B	0.67 (0.48-0.94)	2.01E-02	0.76 (0.55-1.05)	9.69E-02
VLDL - free cholesterol	0.63 (0.42-0.94)	2.27E-02	0.54 (0.36-0.81)	2.68E-03
HDL3 - free cholesterol	0.67 (0.47-0.95)	2.36E-02	0.69 (0.49-0.96)	2.59E-02
LDL6 - cholesterol	0.67 (0.47-0.95)	2.40E-02	0.88 (0.65-1.18)	3.80E-01
VLDL2 - triglycerides	0.62 (0.41-0.95)	2.80E-02	0.73 (0.51-1.05)	9.23E-02
HDL4 - triglycerides	0.66 (0.46-0.96)	2.81E-02	0.69 (0.49-0.96)	2.98E-02
VLDL - phospholipids	0.66 (0.45-0.96)	3.10E-02	0.6 (0.4-0.89)	1.18E-02
LDL - cholesterol	0.68 (0.47-0.97)	3.59E-02	0.79 (0.58-1.08)	1.44E-01
LDL6 - phospholipids	0.69 (0.48-0.98)	3.71E-02	0.88 (0.65-1.18)	3.88E-01
VLDL1 - free cholesterol	0.65 (0.44-0.98)	3.97E-02	0.53 (0.34-0.82)	4.49E-03
VLDL2 - phospholipids	0.65 (0.43-0.98)	3.98E-02	0.77 (0.55-1.07)	1.22E-01
VLDL - triglycerides	0.67 (0.45-0.98)	4.13E-02	0.6 (0.4-0.9)	1.46E-02
VLDL2 - cholesterol	0.61 (0.38-0.98)	4.17E-02	0.66 (0.45-0.96)	3.10E-02
Total plasma - triglycerides	0.67 (0.45-0.99)	4.35E-02	0.58 (0.39-0.86)	7.12E-03
VLDL1 - phospholipids	0.67 (0.45-0.99)	4.48E-02	0.63 (0.43-0.92)	1.80E-02
VLDL1 - triglycerides	0.67 (0.46-0.99)	4.49E-02	0.56 (0.37-0.85)	5.79E-03
VLDL3 - triglycerides	0.67 (0.45-1)	5.19E-02	0.8 (0.56-1.14)	2.22E-01

LDL - phospholipids	0.71 (0.5-1.01)	5.50E-02	0.8 (0.58-1.09)	1.55E-01
LDL6 - free cholesterol	0.72 (0.52-1.01)	5.54E-02	0.97 (0.73-1.3)	8.61E-01
Total plasma - Apo A-1	0.71 (0.5-1.01)	6.02E-02	0.74 (0.55-1.01)	5.44E-02
VLDL - cholesterol	0.68 (0.45-1.02)	6.11E-02	0.58 (0.4-0.86)	6.26E-03
LDL5 - cholesterol	0.72 (0.51-1.02)	6.44E-02	0.79 (0.58-1.07)	1.27E-01
LDL5 - free cholesterol	0.71 (0.49-1.02)	6.54E-02	0.85 (0.63-1.14)	2.76E-01
LDL - Apo B	0.72 (0.51-1.02)	6.71E-02	0.83 (0.61-1.13)	2.33E-01
LDL5 - phospholipids	0.72 (0.51-1.02)	6.83E-02	0.81 (0.59-1.1)	1.73E-01
VLDL1 - cholesterol	0.68 (0.44-1.04)	7.50E-02	0.55 (0.38-0.8)	1.46E-03
VLDL - Apo B	0.72 (0.5-1.04)	7.67E-02	0.7 (0.5-0.99)	4.67E-02
VLDL6 - triglycerides	1.36 (0.97-1.92)	7.73E-02	1.1 (0.77-1.56)	6.11E-01
HDL3 - cholesterol	0.73 (0.52-1.04)	7.83E-02	0.59 (0.43-0.82)	1.80E-03
HDL - Apo A-1	0.73 (0.51-1.04)	8.04E-02	0.73 (0.54-0.99)	4.56E-02
VLDL2 - free cholesterol	0.69 (0.46-1.05)	8.11E-02	0.71 (0.5-1.01)	5.36E-02
LDL5 - Apo B	0.74 (0.52-1.06)	9.87E-02	0.83 (0.61-1.12)	2.18E-01
VLDL4 - triglycerides	0.74 (0.52-1.06)	9.89E-02	0.78 (0.53-1.15)	2.05E-01
IDL - free cholesterol	0.71 (0.47-1.07)	1.02E-01	0.75 (0.55-1.03)	7.45E-02
LDL6 - triglycerides	0.72 (0.49-1.07)	1.03E-01	1.02 (0.72-1.45)	9.23E-01
LDL - free cholesterol	0.76 (0.54-1.06)	1.04E-01	0.87 (0.64-1.18)	3.56E-01
VLDL5 - phospholipids	0.75 (0.53-1.06)	1.05E-01	0.66 (0.49-0.88)	4.73E-03
VLDL5 - cholesterol	0.73 (0.5-1.07)	1.06E-01	0.67 (0.49-0.9)	7.96E-03
VLDL3 - phospholipids	0.74 (0.51-1.07)	1.06E-01	0.81 (0.58-1.13)	2.15E-01
HDL1 - Apo A-2	1.29 (0.94-1.76)	1.10E-01	1.04 (0.77-1.39)	8.02E-01
HDL3 - Apo A-1	0.76 (0.54-1.07)	1.14E-01	0.58 (0.42-0.79)	5.74E-04
IDL - triglycerides	0.7 (0.44-1.1)	1.19E-01	0.47 (0.29-0.78)	3.12E-03
VLDL3 - cholesterol	0.72 (0.48-1.09)	1.20E-01	0.78 (0.55-1.1)	1.56E-01
LDL4 - cholesterol	0.77 (0.56-1.07)	1.21E-01	0.71 (0.51-0.99)	4.55E-02
HDL3 - Apo A-2	0.75 (0.52-1.09)	1.27E-01	0.6 (0.42-0.84)	3.60E-03
LDL1 - cholesterol	0.78 (0.56-1.08)	1.31E-01	0.75 (0.54-1.03)	7.74E-02
VLDL4 - cholesterol	0.75 (0.51-1.09)	1.32E-01	0.71 (0.49-1.03)	7.31E-02
VLDL4 - phospholipids	0.77 (0.54-1.09)	1.42E-01	0.72 (0.49-1.05)	9.15E-02
VLDL3 - free cholesterol	0.75 (0.51-1.1)	1.45E-01	0.76 (0.53-1.08)	1.22E-01
VLDL5 - free cholesterol	0.74 (0.49-1.11)	1.47E-01	0.7 (0.51-0.95)	2.30E-02
VLDL4 - free cholesterol	0.78 (0.56-1.1)	1.58E-01	0.69 (0.48-0.98)	3.70E-02
LDL4 - phospholipids	0.79 (0.57-1.1)	1.58E-01	0.72 (0.52-0.99)	4.05E-02
IDL - phospholipids	0.78 (0.55-1.11)	1.65E-01	0.62 (0.43-0.89)	9.15E-03
VLDL5 - triglycerides	0.78 (0.55-1.11)	1.66E-01	0.66 (0.48-0.9)	8.74E-03
IDL - cholesterol	0.76 (0.51-1.13)	1.75E-01	0.79 (0.58-1.09)	1.50E-01
LDL4 - free cholesterol	0.8 (0.58-1.11)	1.80E-01	0.73 (0.53-1.01)	5.51E-02
HDL3 - phospholipids	0.79 (0.56-1.12)	1.88E-01	0.54 (0.38-0.78)	1.05E-03
LDL1 - phospholipids	0.8 (0.57-1.12)	1.89E-01	0.77 (0.56-1.06)	1.06E-01
HDL1 - phospholipids	1.24 (0.89-1.72)	2.00E-01	1.05 (0.8-1.39)	7.27E-01
HDL1 - Apo A-1	1.23 (0.88-1.72)	2.22E-01	1.08 (0.83-1.41)	5.69E-01
HDL - cholesterol	0.82 (0.59-1.14)	2.40E-01	0.77 (0.56-1.05)	1.02E-01
HDL - free cholesterol	0.82 (0.58-1.15)	2.49E-01	0.85 (0.63-1.14)	2.69E-01

LDL2 - cholesterol	0.82 (0.59-1.16)	2.62E-01	1.09 (0.81-1.45)	5.78E-01
IDL - Apo B	0.82 (0.57-1.17)	2.77E-01	0.82 (0.6-1.14)	2.34E-01
LDL3 - cholesterol	0.83 (0.6-1.16)	2.81E-01	0.95 (0.71-1.28)	7.43E-01
HDL1 - triglycerides	1.18 (0.87-1.59)	2.83E-01	1.02 (0.75-1.37)	9.18E-01
LDL4 - Apo B	0.84 (0.61-1.16)	2.87E-01	0.76 (0.56-1.04)	8.34E-02
LDL2 - triglycerides	1.18 (0.87-1.58)	2.88E-01	1.12 (0.83-1.52)	4.64E-01
LDL2 - free cholesterol	0.84 (0.61-1.17)	3.03E-01	1.1 (0.82-1.48)	5.09E-01
LDL2 - phospholipids	0.84 (0.6-1.18)	3.12E-01	1.08 (0.81-1.45)	6.02E-01
LDL2 - Apo B	0.84 (0.6-1.18)	3.15E-01	1.09 (0.82-1.47)	5.46E-01
LDL3 - phospholipids	0.85 (0.61-1.19)	3.34E-01	0.96 (0.71-1.3)	7.81E-01
LDL1 - Apo B	0.86 (0.61-1.19)	3.54E-01	0.8 (0.58-1.11)	1.87E-01
LDL3 - triglycerides	1.14 (0.84-1.55)	3.96E-01	0.93 (0.68-1.28)	6.61E-01
HDL - phospholipids	0.87 (0.62-1.22)	4.20E-01	0.72 (0.51-1)	5.11E-02
LDL3 - Apo B	0.88 (0.63-1.23)	4.53E-01	1 (0.74-1.36)	9.97E-01
LDL1 - free cholesterol	0.89 (0.66-1.22)	4.69E-01	0.82 (0.59-1.13)	2.14E-01
HDL1 - cholesterol	1.13 (0.81-1.58)	4.73E-01	1.05 (0.8-1.38)	7.16E-01
HDL2 - Apo A-2	1.11 (0.82-1.5)	4.97E-01	0.76 (0.56-1.04)	8.78E-02
LDL3 - free cholesterol	0.91 (0.67-1.23)	5.37E-01	0.98 (0.72-1.34)	9.09E-01
HDL2 - triglycerides	1.09 (0.81-1.47)	5.58E-01	0.8 (0.55-1.15)	2.25E-01
HDL1 - free cholesterol	1.08 (0.76-1.52)	6.67E-01	1.04 (0.79-1.38)	7.62E-01
LDL4 - triglycerides	1.06 (0.79-1.44)	6.89E-01	1.05 (0.77-1.45)	7.42E-01
HDL2 - Apo A-1	1.05 (0.77-1.45)	7.47E-01	0.77 (0.56-1.07)	1.15E-01
HDL3 - triglycerides	0.95 (0.69-1.31)	7.53E-01	0.67 (0.45-0.98)	3.85E-02
LDL5 - triglycerides	0.95 (0.68-1.33)	7.71E-01	1.03 (0.75-1.43)	8.37E-01
HDL2 - cholesterol	0.96 (0.69-1.33)	8.01E-01	0.75 (0.54-1.03)	7.74E-02
LDL1 - triglycerides	0.97 (0.7-1.36)	8.78E-01	1.01 (0.72-1.41)	9.60E-01
VLDL6 - free cholesterol	0.98 (0.73-1.3)	8.82E-01	0.56 (0.38-0.82)	3.30E-03
LDL - triglycerides	0.99 (0.7-1.38)	9.32E-01	1.14 (0.79-1.64)	4.80E-01
HDL - triglycerides	0.99 (0.71-1.36)	9.34E-01	0.75 (0.52-1.09)	1.36E-01
HDL2 - free cholesterol	0.99 (0.7-1.4)	9.49E-01	0.84 (0.61-1.15)	2.72E-01
HDL2 - phospholipids	1 (0.73-1.38)	9.96E-01	0.7 (0.49-0.98)	4.01E-02

Table S4 – Linear regression models of HDL-4-Apo A-2 and plasma proteins measurement in the combined discovery and validation cohort adjusting for confounding factors including age, gender, ethnicity, diabetes mellitus, statin therapy, oral anticoagulation treatment, renal and hepatic impairment, as well as survival status and batch. In addition, corresponding spearman's correlation in the combined discovery and validation cohort. Marked in grey are the 10 proteins which met Bonferroni correction ($p<4.5 \times 10^{-5}$).

**Plasma proteome**	**Combined discovery and validation cohort**				
	**Linear regression model adj. for potential confounding factors. survival status and batch**			**Spearman 's correlation**	
	Beta	SE	p value	rho	p value
Prekallikrein	0.59	0.10	4.86E-08	0.555	1.11E-15
Apo A I	0.68	0.12	2.08E-07	0.460	1.15E-10
a2 Antiplasmin	0.51	0.09	3.38E-07	0.509	4.50E-13
CNDP1	0.69	0.13	4.58E-07	0.493	3.13E-12
Coagulation Factor XI	0.69	0.13	6.12E-07	0.487	6.33E-12
Afamin	0.59	0.13	1.07E-05	-0.474	2.65E-11
NRP1	-0.50	0.11	1.28E-05	0.465	6.71E-11
Growth hormone receptor	0.43	0.09	1.37E-05	0.487	5.96E-12
a2 HS Glycoprotein	0.42	0.09	2.04E-05	0.365	5.83E-07
P Cadherin	0.46	0.11	8.25E-05	0.435	1.59E-09
ERBB3	0.96	0.24	9.56E-05	0.413	1.13E-08
PCI	0.55	0.14	1.44E-04	0.417	7.80E-09
LYVE1	-0.44	0.11	2.16E-04	0.444	5.82E-10
Plasminogen	0.43	0.11	2.17E-04	-0.341	3.32E-06
ATS13	0.90	0.24	3.03E-04	0.422	5.47E-09
Ficolin 3	0.49	0.14	4.12E-04	-0.415	9.63E-09
Notch 3	-0.45	0.13	6.76E-04	-0.439	9.50E-10
Kallistatin	0.38	0.11	6.99E-04	0.400	3.39E-08
TAFI	0.35	0.10	7.04E-04	0.394	5.63E-08
BMP 1	0.38	0.11	8.78E-04	0.320	1.45E-05
TIMP 1	-0.33	0.10	9.88E-04	0.455	2.30E-10
Kallikrein 7	0.34	0.10	1.01E-03	-0.424	4.11E-09
TIMP 2	-0.36	0.11	1.22E-03	0.323	1.16E-05
SAP	0.44	0.13	1.26E-03	0.333	6.07E-06
Factor I	0.28	0.09	1.30E-03	-0.415	9.33E-09
Carbonic anhydrase 6	0.36	0.11	1.73E-03	0.331	7.03E-06
RET	0.28	0.09	1.75E-03	0.438	1.23E-09
ATS15	1.06	0.34	2.21E-03	-0.252	7.54E-04

ERBB1	0.32	0.10	2.36E-03	0.439	9.59E-10
Haptoglobin Mixed Type	-0.61	0.20	2.42E-03	0.362	7.28E-07
IL 1 R AcP	0.30	0.10	2.97E-03	0.399	3.75E-08
paraoxonase 1	0.77	0.26	3.28E-03	-0.239	1.36E-03
Integrin a1b1	-0.25	0.08	3.51E-03	0.369	4.26E-07
IGFBP 2	-0.28	0.10	3.62E-03	-0.150	4.66E-02
LG3BP	0.51	0.17	3.93E-03	0.332	6.54E-06
Angiostatin	0.30	0.10	4.27E-03	-0.374	3.26E-07
FETUB	0.10	0.04	4.92E-03	-0.322	1.35E-05
IP 10	-0.64	0.22	5.17E-03	-0.282	1.52E-04
C6	0.40	0.14	5.26E-03	0.204	6.50E-03
DC SIGNR	0.26	0.09	5.47E-03	-0.263	4.16E-04
Endothelin converting enzyme 1	0.27	0.10	6.32E-03	-0.318	1.66E-05
Apo E	0.32	0.12	6.52E-03	-0.324	1.15E-05
NCAM L1	0.57	0.21	6.61E-03	0.384	1.40E-07
HRG	0.42	0.15	7.01E-03	0.195	9.20E-03
ECM1	-0.30	0.11	7.16E-03	0.207	5.68E-03
Catalase	0.21	0.08	7.26E-03	0.303	4.10E-05
Lymphotactin	1.18	0.44	7.60E-03	-0.266	3.70E-04
Factor B	0.35	0.13	7.69E-03	0.282	1.40E-04
SPINT2	-0.30	0.11	9.18E-03	-0.208	5.59E-03
Glypican 3	0.40	0.15	9.45E-03	-0.098	1.95E-01
DR6	0.69	0.26	9.53E-03	-0.268	3.03E-04
Fibrinogen g chain dimer	-0.32	0.12	9.90E-03	0.333	6.38E-06
Cystatin M	0.26	0.10	9.91E-03	-0.416	9.40E-09
HSP 70	0.60	0.23	1.06E-02	0.328	8.66E-06
ALCAM	0.77	0.30	1.08E-02	-0.360	9.09E-07
Cadherin 5	0.74	0.29	1.10E-02	-0.270	3.03E-04
DR3	-0.29	0.11	1.15E-02	0.199	7.83E-03
Thymidine kinase	0.38	0.15	1.17E-02	0.326	1.04E-05
D dimer	-0.30	0.12	1.22E-02	0.170	2.36E-02
tPA	0.44	0.17	1.22E-02	-0.286	1.11E-04
TNFSF18	0.21	0.08	1.27E-02	-0.234	1.78E-03
ENTP5	0.29	0.11	1.34E-02	0.294	6.95E-05
IR	0.48	0.19	1.50E-02	-0.288	1.07E-04
Lymphotoxin a1 b2	0.28	0.11	1.54E-02	-0.312	2.45E-05
Apo E3	0.26	0.11	1.60E-02	-0.101	1.83E-01
M CSF R	-0.36	0.15	1.62E-02	-0.201	7.44E-03
X4 1BB ligand	0.46	0.19	1.77E-02	0.199	8.19E-03
MFGM	0.21	0.09	1.78E-02	0.241	1.25E-03
TLR4 MD 2 complex	0.55	0.23	1.84E-02	-0.207	5.81E-03
CD27	0.30	0.13	1.85E-02	0.305	3.63E-05
Angiotensinogen	0.25	0.10	1.93E-02	0.324	1.17E-05
VEGF sR2	0.23	0.10	1.95E-02	0.275	2.11E-04
FSH	0.15	0.06	1.97E-02	-0.223	2.79E-03

Apo E4	0.24	0.10	1.98E-02	0.101	1.83E-01
gp130 soluble	0.78	0.33	2.02E-02	0.339	4.20E-06
Coagulation Factor V	0.28	0.12	2.03E-02	0.242	1.18E-03
BGH3	0.71	0.30	2.04E-02	0.227	2.37E-03
SREC I	-0.22	0.10	2.31E-02	-0.300	5.58E-05
Apo E2	0.26	0.11	2.32E-02	0.140	6.24E-02
ERAB	-0.27	0.12	2.35E-02	-0.174	2.07E-02
CK MM	0.23	0.10	2.36E-02	0.305	3.58E-05
NRX3B	0.55	0.24	2.37E-02	0.125	9.87E-02
ART	0.39	0.17	2.39E-02	0.194	9.55E-03
GCP 2	-0.31	0.14	2.47E-02	-0.417	8.59E-09
eIF 5	-0.41	0.18	2.47E-02	-0.275	2.18E-04
BCAR3	0.67	0.29	2.50E-02	0.318	1.59E-05
sTie 1	0.61	0.27	2.56E-02	0.350	1.78E-06
Hat1	-0.21	0.09	2.60E-02	0.336	4.84E-06
NR1D1	0.43	0.19	2.64E-02	-0.104	1.70E-01
sTie 2	0.65	0.29	2.66E-02	-0.127	9.48E-02
ADAMTS 4	0.23	0.10	2.73E-02	0.239	2.35E-03
Cadherin E	0.23	0.10	2.74E-02	0.284	1.25E-04
PCSK9	0.21	0.09	2.86E-02	-0.136	7.02E-02
X6Ckine	0.72	0.32	2.86E-02	-0.175	2.01E-02
IL 1F7	0.24	0.11	2.96E-02	0.133	7.85E-02
Antithrombin III	0.29	0.13	3.08E-02	0.216	4.10E-03
IL 27	0.25	0.11	3.10E-02	-0.279	1.66E-04
Thrombospondin 1	-0.18	0.08	3.12E-02	-0.197	8.76E-03
VEGF sR3	0.52	0.24	3.14E-02	-0.101	1.83E-01
CLM6	-0.65	0.30	3.23E-02	0.262	4.35E-04
Kallikrein 6	0.20	0.09	3.27E-02	-0.202	7.05E-03
Properdin	0.26	0.12	3.39E-02	-0.097	2.02E-01
CDK2 cyclin A	0.25	0.12	3.40E-02	-0.200	7.60E-03
STK16	0.18	0.09	3.48E-02	0.311	2.62E-05
MIS	0.19	0.09	3.49E-02	-0.263	4.20E-04
Cyclophilin F	-0.17	0.08	3.64E-02	-0.309	3.06E-05
IGF I sR	0.42	0.20	3.79E-02	-0.130	8.57E-02
ILT 2	0.34	0.16	3.79E-02	-0.073	3.34E-01
Cathepsin B	-0.64	0.31	3.80E-02	0.189	1.16E-02
b ECGF	0.19	0.09	3.95E-02	0.205	6.44E-03
HO 2	-0.17	0.08	4.01E-02	-0.163	3.05E-02
PSA	0.21	0.10	4.03E-02	-0.226	2.54E-03
FUT5	-0.17	0.08	4.07E-02	0.217	3.78E-03
MK08	-0.21	0.10	4.23E-02	0.255	6.37E-04
FGF 17	0.19	0.09	4.24E-02	-0.206	5.94E-03
Chk2	0.32	0.16	4.36E-02	-0.159	3.46E-02
Factor H	0.25	0.12	4.45E-02	-0.260	5.02E-04
TMA	0.20	0.10	4.45E-02	0.196	9.25E-03

CATC	0.19	0.10	4.47E-02	0.218	3.49E-03
SDF 1	-0.21	0.11	4.58E-02	0.134	7.57E-02
Eotaxin 2	0.28	0.14	4.61E-02	0.152	4.45E-02
Calpastatin	0.19	0.10	4.69E-02	-0.129	8.68E-02
BSSP4	0.37	0.19	4.84E-02	-0.158	3.67E-02
FN1 3	-0.25	0.12	4.89E-02	0.211	4.73E-03
NET4	-0.23	0.12	4.92E-02	-0.194	9.78E-03
Angiopoietin 2	-0.27	0.14	4.93E-02	0.074	3.29E-01
Bone proteoglycan II	-0.27	0.14	4.98E-02	-0.185	1.38E-02
Kininogen HMW	0.19	0.10	5.00E-02	0.215	4.08E-03
CK MB	0.19	0.10	5.09E-02	0.251	7.74E-04
MP2K4	0.27	0.14	5.15E-02	0.175	2.04E-02
IL 10	0.51	0.26	5.39E-02	-0.175	2.04E-02
Siglec 3	0.33	0.17	5.39E-02	0.144	5.57E-02
MASP3	0.19	0.10	5.44E-02	0.324	1.15E-05
Aminoacylase 1	0.37	0.19	5.61E-02	0.192	1.04E-02
NID2	-0.15	0.08	5.63E-02	0.163	3.08E-02
Sonic Hedgehog	0.19	0.10	5.63E-02	0.191	1.09E-02
SLAF6	-0.19	0.10	5.81E-02	-0.300	5.43E-05
CNTF	0.20	0.11	5.96E-02	-0.256	6.20E-04
X41	-0.52	0.27	6.02E-02	0.162	3.16E-02
Neurotrophin 3	-0.21	0.11	6.04E-02	0.213	4.47E-03
TSP4	-0.22	0.12	6.09E-02	-0.046	5.41E-01
G CSF	0.17	0.09	6.21E-02	0.182	1.51E-02
Collectin Kidney 1	0.34	0.18	6.27E-02	-0.197	8.84E-03
TNF sR II	-0.68	0.36	6.28E-02	-0.265	3.88E-04
Desmoglein 2	0.72	0.39	6.30E-02	-0.056	4.58E-01
PAK3	0.18	0.10	6.37E-02	0.221	3.33E-03
IgG	0.83	0.44	6.40E-02	0.077	3.10E-01
RTN4	0.79	0.42	6.47E-02	-0.154	4.10E-02
HIV 2 Rev	0.13	0.07	6.70E-02	0.244	1.08E-03
CD70	0.17	0.09	6.82E-02	-0.104	1.67E-01
JAG1	0.57	0.31	6.86E-02	0.251	7.71E-04
TLR2	0.61	0.33	6.87E-02	0.143	5.72E-02
Angiopoietin 1	-0.15	0.08	6.92E-02	0.274	2.28E-04
OBCAM	0.16	0.09	7.09E-02	0.019	8.02E-01
IL 17B	0.20	0.11	7.10E-02	0.271	2.61E-04
Apo D	0.15	0.08	7.11E-02	0.016	8.31E-01
IFN aA	0.23	0.13	7.11E-02	-0.130	8.40E-02
LAG 3	-0.41	0.22	7.20E-02	0.098	1.95E-01
TEC	-0.16	0.09	7.23E-02	0.209	5.32E-03
Leptin	0.17	0.10	7.44E-02	-0.114	1.32E-01
HSP 60	-0.54	0.30	7.54E-02	0.188	1.20E-02
Protein disulfide isomerase A3	-0.17	0.09	7.55E-02	0.262	4.27E-04
Histone H2A z	-0.19	0.10	7.55E-02	0.105	1.64E-01

RASA1	0.17	0.10	7.58E-02	-0.298	5.77E-05
AURKB	0.15	0.08	7.72E-02	0.231	1.99E-03
Nogo Receptor	0.42	0.24	7.79E-02	0.124	1.01E-01
I 309	0.16	0.09	7.84E-02	0.166	2.75E-02
Trypsin	0.41	0.23	7.94E-02	0.194	9.76E-03
PIM1	0.17	0.09	7.94E-02	-0.136	7.10E-02
Livin B	0.22	0.13	7.98E-02	-0.203	6.67E-03
Prothrombin	0.23	0.13	8.01E-02	0.261	4.49E-04
annexin VI	-0.42	0.24	8.06E-02	0.077	3.11E-01
DAPK2	-0.17	0.10	8.21E-02	0.221	3.25E-03
ASAH2	0.17	0.09	8.21E-02	-0.090	2.33E-01
IL 20	0.19	0.11	8.27E-02	-0.217	3.73E-03
OPG	0.16	0.09	8.41E-02	-0.076	3.16E-01
VEGF C	-0.17	0.10	8.44E-02	0.237	1.52E-03
sICAM 1	-0.45	0.26	8.51E-02	-0.127	9.21E-02
SCGF beta	0.54	0.31	8.60E-02	-0.176	1.95E-02
C8	0.15	0.09	8.76E-02	-0.216	4.15E-03
CDON	0.19	0.11	8.80E-02	0.178	1.81E-02
IgE	-0.16	0.10	8.84E-02	-0.197	8.78E-03
ADAM 9	-0.16	0.09	8.85E-02	-0.160	3.35E-02
Soggy 1	0.34	0.20	8.93E-02	-0.246	9.63E-04
TrATPase	0.20	0.12	8.97E-02	-0.202	7.20E-03
Marapsin	0.19	0.11	9.06E-02	0.005	9.43E-01
CRIS3	0.24	0.14	9.10E-02	-0.102	1.77E-01
Tropomyosin 2	0.17	0.10	9.23E-02	-0.235	1.70E-03
C9	-0.17	0.10	9.27E-02	-0.076	3.17E-01
ARGI1	0.16	0.09	9.31E-02	0.202	7.25E-03
MIP 1a	-0.21	0.12	9.32E-02	-0.169	2.42E-02
TNFSF15	-0.51	0.30	9.36E-02	0.135	7.43E-02
Activin A	0.26	0.15	9.48E-02	-0.161	3.23E-02
Protein C	0.33	0.20	9.49E-02	-0.373	3.68E-07
PKC B II	-0.29	0.17	9.51E-02	0.044	5.61E-01
FGF 10	0.16	0.10	9.54E-02	-0.046	5.44E-01
CSK	-0.29	0.17	9.62E-02	0.204	6.59E-03
HCC 1	-0.16	0.10	9.95E-02	0.187	1.30E-02
kallikrein 13	0.14	0.09	1.00E-01	0.151	4.50E-02
MIA	0.18	0.11	1.02E-01	0.274	2.39E-04
BMP 14	0.47	0.28	1.04E-01	-0.098	1.96E-01
MCP 2	-0.53	0.32	1.05E-01	0.140	6.29E-02
Survivin	0.15	0.09	1.05E-01	-0.225	2.62E-03
FCN1	0.20	0.12	1.05E-01	-0.109	1.47E-01
Dtk	0.17	0.10	1.06E-01	-0.184	1.44E-02
sCD163	0.32	0.20	1.06E-01	0.178	1.83E-02
TGF b R II	0.32	0.20	1.08E-01	-0.183	1.54E-02
Carbonic Anhydrase X	0.14	0.09	1.08E-01	-0.102	1.78E-01

RANK	-0.11	0.07	1.09E-01	0.228	2.30E-03
X4EBP2	-0.12	0.08	1.09E-01	0.243	1.14E-03
Galectin 4	-0.37	0.23	1.10E-01	-0.105	1.66E-01
MK12	-0.16	0.10	1.11E-01	-0.287	1.09E-04
CD226	-0.13	0.08	1.11E-01	0.155	4.06E-02
MMP 1	-0.16	0.10	1.12E-01	0.250	7.79E-04
EPI	-0.15	0.10	1.13E-01	-0.179	1.77E-02
AK1A1	-0.14	0.09	1.17E-01	-0.073	3.36E-01
Fibrinogen	-0.22	0.14	1.18E-01	-0.195	9.16E-03
Thyroxine Binding Globulin	0.17	0.11	1.19E-01	0.160	3.40E-02
ASAHL	0.20	0.13	1.20E-01	0.198	8.28E-03
p27Kip1	-0.15	0.10	1.21E-01	0.139	6.65E-02
CYTF	-0.14	0.09	1.22E-01	0.184	1.46E-02
Transferrin	0.18	0.11	1.22E-01	-0.070	3.53E-01
TRY3	0.47	0.30	1.23E-01	-0.134	7.78E-02
WIF 1	0.39	0.25	1.25E-01	0.255	6.34E-04
Apo B	0.15	0.09	1.25E-01	0.168	2.63E-02
Angiogenin	0.51	0.33	1.25E-01	0.210	5.17E-03
LYNB	-0.39	0.25	1.26E-01	-0.063	4.05E-01
MCP 3	-0.18	0.12	1.27E-01	0.050	5.06E-01
sL Selectin	-0.66	0.43	1.27E-01	0.185	1.40E-02
discoidin domain receptor 1	0.51	0.33	1.27E-01	0.171	2.30E-02
Tropomyosin 1 alpha chain	0.10	0.06	1.30E-01	-0.231	2.07E-03
RAP	-0.34	0.22	1.31E-01	-0.239	1.35E-03
Glucagon	-0.14	0.09	1.31E-01	-0.151	4.42E-02
BLC	-0.40	0.26	1.32E-01	0.147	5.09E-02
Met	0.15	0.10	1.33E-01	-0.160	3.41E-02
Carbonic Anhydrase IV	0.15	0.10	1.34E-01	0.153	4.26E-02
sRAGE	0.14	0.10	1.35E-01	0.135	7.38E-02
kallikrein 14	0.13	0.08	1.36E-01	0.208	5.47E-03
K ras	0.13	0.09	1.37E-01	-0.124	1.02E-01
HSP70 protein 8	0.15	0.10	1.37E-01	-0.191	1.09E-02
MK01	-0.13	0.09	1.38E-01	0.232	1.96E-03
PSME3	0.33	0.22	1.39E-01	-0.061	4.21E-01
FSTL3	-0.14	0.09	1.39E-01	-0.133	7.76E-02
IL 10 Rb	0.17	0.11	1.39E-01	-0.200	7.58E-03
NMT1	-0.15	0.10	1.40E-01	0.332	6.37E-06
CBX5	0.16	0.11	1.40E-01	-0.060	4.26E-01
RAD51	0.14	0.09	1.40E-01	0.259	5.09E-04
C7	0.53	0.36	1.41E-01	0.117	1.20E-01
Aggrecan	0.14	0.10	1.41E-01	0.228	2.30E-03
BNP 32	-0.14	0.09	1.41E-01	0.174	2.11E-02
PCNA	0.33	0.23	1.42E-01	0.151	4.61E-02
Mn SOD	0.14	0.10	1.42E-01	-0.255	6.72E-04
Ephrin A5	0.17	0.12	1.43E-01	0.155	3.93E-02

DLL1	-0.19	0.13	1.44E-01	-0.081	2.82E-01
uPA	-0.13	0.09	1.45E-01	0.109	1.50E-01
ING1	-0.13	0.09	1.46E-01	-0.087	2.54E-01
JAK2	0.13	0.09	1.47E-01	-0.161	3.31E-02
Ferritin	-0.17	0.12	1.47E-01	-0.142	5.88E-02
Nidogen	-0.13	0.09	1.49E-01	0.320	1.45E-05
PDE11	-0.13	0.09	1.49E-01	0.254	6.37E-04
Lymphotoxin a2 b1	0.12	0.09	1.50E-01	-0.094	2.12E-01
HIPK3	0.16	0.11	1.50E-01	-0.136	7.11E-02
a1 Antitrypsin	-0.32	0.22	1.50E-01	-0.052	4.89E-01
PCSK7	0.17	0.11	1.50E-01	0.030	6.89E-01
Lymphotoxin b R	0.13	0.09	1.51E-01	0.180	1.69E-02
PYY	-0.16	0.11	1.51E-01	-0.144	5.67E-02
MPIF 1	0.43	0.30	1.51E-01	-0.062	4.09E-01
MMP 12	-0.28	0.19	1.51E-01	0.097	2.01E-01
IL 3 Ra	-0.14	0.10	1.52E-01	-0.099	1.90E-01
HCG	0.13	0.09	1.53E-01	0.131	8.23E-02
EP15R	-0.12	0.08	1.53E-01	0.101	1.84E-01
Adiponectin	-0.17	0.12	1.55E-01	-0.057	4.55E-01
granzyme A	-0.34	0.24	1.56E-01	-0.100	1.88E-01
Notch 1	0.13	0.09	1.56E-01	-0.073	3.31E-01
Persephin	0.14	0.10	1.57E-01	-0.132	8.19E-02
prostatic binding protein	-0.11	0.07	1.58E-01	-0.136	7.03E-02
suPAR	0.35	0.25	1.58E-01	-0.071	3.45E-01
PSA2	0.17	0.12	1.59E-01	0.145	5.38E-02
Activin RIB	0.15	0.10	1.59E-01	0.252	7.21E-04
IL 17E	0.13	0.09	1.59E-01	-0.108	1.52E-01
CD39	0.14	0.10	1.60E-01	-0.112	1.39E-01
AMPM2	-0.14	0.10	1.62E-01	-0.188	1.25E-02
Lipocalin 2	-0.57	0.41	1.62E-01	-0.092	2.25E-01
TIG2	0.60	0.42	1.64E-01	0.020	7.90E-01
TIMD3	-0.15	0.10	1.64E-01	-0.275	2.23E-04
GPVI	-0.12	0.08	1.65E-01	0.158	3.57E-02
HPLN1	0.13	0.09	1.65E-01	0.171	2.29E-02
Activin AB	-0.19	0.13	1.65E-01	0.179	1.71E-02
EphA1	0.16	0.11	1.66E-01	-0.221	3.18E-03
ETHE1	0.11	0.08	1.66E-01	-0.200	7.91E-03
IDUA	0.13	0.10	1.66E-01	0.218	3.52E-03
Olfactomedin 4	0.19	0.14	1.67E-01	-0.107	1.58E-01
sRANKL	0.13	0.09	1.67E-01	-0.080	2.90E-01
CAMK2B	-0.13	0.10	1.70E-01	0.178	1.81E-02
DC SIGN	-0.10	0.07	1.71E-01	0.089	2.37E-01
X4 1BB	-0.33	0.24	1.71E-01	-0.027	7.20E-01
Albumin	-1.81	1.32	1.72E-01	0.155	4.01E-02
CRP	-0.10	0.07	1.72E-01	-0.123	1.02E-01

ZAP70	0.14	0.10	1.73E-01	-0.259	5.03E-04
GREM1	-0.15	0.11	1.74E-01	-0.138	6.76E-02
GDF 9	-0.13	0.10	1.74E-01	0.189	1.21E-02
IL 34	0.14	0.10	1.75E-01	-0.080	2.90E-01
DKK1	-0.15	0.11	1.77E-01	-0.088	2.44E-01
CLC4K	0.11	0.08	1.77E-01	0.166	2.73E-02
ULBP 3	-0.13	0.09	1.77E-01	-0.103	1.73E-01
Aflatoxin B1 aldehyde reductase	-0.11	0.08	1.78E-01	0.161	3.32E-02
LRIG3	0.13	0.10	1.78E-01	-0.096	2.02E-01
CCL28	-0.17	0.13	1.78E-01	-0.224	2.73E-03
FYN	-0.13	0.10	1.79E-01	0.211	4.90E-03
NCC27	-0.12	0.09	1.80E-01	0.062	4.14E-01
CAMK2D	-0.13	0.10	1.80E-01	-0.070	3.54E-01
X3HIDH	0.12	0.09	1.81E-01	0.164	2.99E-02
CSF 1	-0.15	0.11	1.81E-01	-0.106	1.60E-01
PGP9 5	-0.12	0.09	1.82E-01	0.053	4.85E-01
CD40 ligand soluble	-0.15	0.11	1.82E-01	-0.129	8.74E-02
X17 beta HSD 1	0.16	0.12	1.83E-01	0.054	4.75E-01
FGF 6	-0.12	0.09	1.83E-01	-0.113	1.35E-01
CPNE1	-0.11	0.08	1.84E-01	-0.068	3.71E-01
KIRR3	0.13	0.10	1.84E-01	0.181	1.60E-02
Trypsin 2	0.25	0.19	1.85E-01	0.249	8.45E-04
Galectin 3	0.11	0.08	1.87E-01	-0.094	2.15E-01
TCTP	-0.11	0.08	1.88E-01	0.189	1.19E-02
Stanniocalcin 1	0.30	0.23	1.89E-01	0.160	3.40E-02
Thrombopoietin Receptor	0.38	0.29	1.91E-01	0.211	4.82E-03
HDGR2	-0.14	0.11	1.92E-01	0.001	9.93E-01
TPSB2	0.14	0.11	1.92E-01	-0.067	3.80E-01
CD97	0.12	0.09	1.92E-01	0.130	8.43E-02
IL 1a	0.15	0.12	1.93E-01	-0.058	4.43E-01
PAFAH beta subunit	-0.10	0.08	1.94E-01	0.163	3.09E-02
tau	-0.10	0.07	1.94E-01	0.160	3.35E-02
Esterase D	-0.15	0.12	1.94E-01	0.283	1.46E-04
MMP 8	0.28	0.22	1.95E-01	-0.119	1.14E-01
LYN	-0.12	0.09	1.96E-01	-0.058	4.42E-01
SHC1	-0.11	0.09	1.96E-01	0.159	3.54E-02
MAPK14	-0.12	0.09	1.97E-01	-0.098	1.94E-01
AMPK a2b2g1	-0.12	0.10	1.99E-01	0.147	5.11E-02
gpIIbIIIa	-0.16	0.12	1.99E-01	0.168	2.52E-02
SCGF alpha	0.31	0.24	2.00E-01	-0.039	6.11E-01
NCAM 120	0.14	0.11	2.00E-01	-0.092	2.23E-01
ARP19	-0.13	0.10	2.00E-01	-0.082	2.76E-01
CD5L	-0.16	0.12	2.00E-01	0.137	6.95E-02
NACA	-0.10	0.08	2.01E-01	-0.089	2.42E-01
TrkB	0.14	0.11	2.01E-01	0.170	2.45E-02

calgranulin B	0.20	0.16	2.01E-01	0.260	4.74E-04
sICAM 3	0.35	0.27	2.01E-01	0.002	9.83E-01
SBDS	-0.12	0.09	2.02E-01	-0.080	2.91E-01
GFRa 2	-0.13	0.10	2.03E-01	-0.042	5.76E-01
DHH	-0.12	0.10	2.05E-01	0.217	3.87E-03
RSK like protein kinase	-0.12	0.09	2.05E-01	0.046	5.43E-01
ULBP 1	0.15	0.12	2.06E-01	0.264	3.90E-04
HSP 90a b	-0.11	0.09	2.07E-01	-0.115	1.29E-01
CD30	-0.43	0.34	2.07E-01	-0.052	4.92E-01
kallikrein 5	0.14	0.11	2.07E-01	-0.111	1.41E-01
WFKN2	0.28	0.22	2.07E-01	-0.088	2.46E-01
RS3	-0.13	0.11	2.08E-01	-0.084	2.66E-01
Neurotrophin 5	0.13	0.10	2.09E-01	0.064	3.94E-01
CNTN2	0.29	0.23	2.09E-01	-0.087	2.48E-01
DBNL	-0.12	0.09	2.09E-01	-0.069	3.61E-01
Carbonic anhydrase III	0.14	0.11	2.09E-01	-0.129	8.68E-02
AMHR2	-0.15	0.12	2.10E-01	-0.055	4.68E-01
NXPH1	0.12	0.09	2.11E-01	0.021	7.84E-01
CYTN	0.14	0.12	2.11E-01	-0.095	2.08E-01
ALT	0.24	0.19	2.12E-01	0.057	4.47E-01
FGFR4	-0.18	0.15	2.12E-01	-0.062	4.13E-01
JNK2	-0.67	0.53	2.12E-01	-0.095	2.09E-01
FCN2	0.13	0.10	2.13E-01	0.142	6.02E-02
PF 4	-0.11	0.09	2.13E-01	0.004	9.60E-01
EF 1 beta	-0.11	0.09	2.14E-01	-0.101	1.81E-01
HPV E7 Type 16	0.18	0.14	2.14E-01	0.176	1.92E-02
HMGR	0.13	0.10	2.15E-01	-0.029	7.03E-01
SH21A	-0.16	0.13	2.15E-01	-0.049	5.16E-01
GPNMB	-0.48	0.38	2.15E-01	0.200	7.63E-03
BAFF	0.14	0.11	2.16E-01	0.092	2.26E-01
MFRP	0.17	0.14	2.16E-01	-0.073	3.31E-01
PAFAH	-0.38	0.31	2.16E-01	-0.095	2.09E-01
Testican 2	0.18	0.15	2.16E-01	-0.107	1.55E-01
VEGF121	-0.19	0.15	2.17E-01	-0.057	4.52E-01
NAP 2	-0.11	0.09	2.17E-01	-0.038	6.14E-01
PKB a b g	-0.11	0.09	2.18E-01	-0.067	3.72E-01
DLC8	0.09	0.07	2.18E-01	0.100	1.85E-01
CAMK1D	0.11	0.09	2.20E-01	0.016	8.37E-01
Calcineurin	-0.11	0.09	2.21E-01	-0.034	6.50E-01
Ubiquitin	-0.15	0.13	2.23E-01	0.056	4.57E-01
BMPER	0.15	0.12	2.23E-01	-0.023	7.59E-01
X37694	-0.10	0.09	2.24E-01	-0.127	9.32E-02
annexin II	-0.13	0.11	2.24E-01	-0.010	8.97E-01
MAPK2	-0.12	0.10	2.24E-01	-0.047	5.32E-01
KIF23	-0.11	0.09	2.25E-01	-0.090	2.36E-01

Dkk 4	-0.13	0.11	2.25E-01	0.112	1.37E-01
CD22	0.14	0.11	2.26E-01	-0.010	8.93E-01
MK11	-0.16	0.14	2.27E-01	-0.074	3.27E-01
ALK 1	-0.38	0.32	2.27E-01	-0.110	1.47E-01
ARI3A	-0.11	0.09	2.29E-01	0.168	2.59E-02
EPHA3	0.28	0.23	2.29E-01	-0.149	4.89E-02
GRB2 related adapter protein 2	0.14	0.11	2.32E-01	-0.062	4.14E-01
UBC9	-0.09	0.08	2.35E-01	0.118	1.18E-01
CYTT	0.13	0.11	2.35E-01	-0.036	6.30E-01
Lamin B1	-0.12	0.10	2.36E-01	0.012	8.77E-01
Calcineurin B a	0.10	0.08	2.37E-01	0.031	6.85E-01
amyloid precursor protein	-0.10	0.08	2.37E-01	0.152	4.35E-02
TAK1 TAB1	-0.10	0.08	2.37E-01	0.189	1.17E-02
IL 2	0.12	0.10	2.38E-01	0.099	1.92E-01
PTP 1C	-0.12	0.10	2.40E-01	-0.019	7.97E-01
Stress induced phosphoprotein 1	-0.10	0.08	2.40E-01	-0.060	4.25E-01
ERP29	0.42	0.36	2.41E-01	-0.042	5.79E-01
BTK	-0.11	0.09	2.42E-01	-0.249	8.36E-04
CTAP III	-0.10	0.09	2.43E-01	-0.140	6.30E-02
FGF 16	-0.10	0.09	2.44E-01	-0.072	3.42E-01
IL 23 R	-0.11	0.09	2.46E-01	-0.085	2.60E-01
SNAA	-0.09	0.07	2.46E-01	0.028	7.13E-01
GX	0.33	0.28	2.47E-01	-0.076	3.17E-01
b Endorphin	0.14	0.12	2.49E-01	-0.158	3.56E-02
IGFBP 3	0.14	0.12	2.49E-01	-0.083	2.74E-01
HCK	0.25	0.22	2.49E-01	0.216	3.88E-03
SE6L2	-0.10	0.09	2.51E-01	0.208	5.62E-03
PPID	-0.12	0.10	2.51E-01	-0.220	3.33E-03
SAA	-0.11	0.09	2.52E-01	0.135	7.30E-02
sE Selectin	0.29	0.25	2.53E-01	0.020	7.88E-01
CD36 ANTIGEN	0.15	0.13	2.54E-01	-0.061	4.21E-01
AIP	-0.10	0.09	2.54E-01	0.040	6.03E-01
PH	0.11	0.10	2.56E-01	0.062	4.15E-01
DRG 1	-0.09	0.08	2.57E-01	0.059	4.36E-01
PESC	-0.22	0.19	2.57E-01	0.104	1.69E-01
Caspase 3	-0.11	0.10	2.59E-01	-0.234	1.75E-03
TGM3	0.16	0.14	2.59E-01	-0.012	8.73E-01
PTP 1B	-0.10	0.09	2.62E-01	0.105	1.66E-01
CNTFR alpha	0.11	0.10	2.62E-01	0.120	1.11E-01
IGFBP 4	0.09	0.08	2.62E-01	0.144	5.66E-02
BFL1	-0.11	0.09	2.63E-01	0.117	1.21E-01
RELT	0.27	0.24	2.65E-01	0.153	4.30E-02
Moesin	-0.10	0.09	2.65E-01	-0.019	8.03E-01
MSP R	0.13	0.11	2.68E-01	-0.028	7.12E-01
P Selectin	-0.10	0.09	2.69E-01	-0.205	6.21E-03

PIGR	-0.10	0.09	2.69E-01	0.036	6.39E-01
LY86	-0.10	0.09	2.69E-01	0.190	1.13E-02
SKP1	-0.09	0.09	2.71E-01	-0.062	4.15E-01
FCG2A B	-0.18	0.16	2.72E-01	-0.035	6.43E-01
GM CSF	-0.14	0.13	2.73E-01	0.015	8.38E-01
LY9	-0.27	0.24	2.74E-01	-0.026	7.35E-01
Endocan	-0.12	0.11	2.74E-01	-0.099	1.90E-01
NKp44	0.13	0.12	2.75E-01	-0.040	5.95E-01
PDE7A	0.17	0.16	2.76E-01	-0.057	4.51E-01
ERK 1	-0.10	0.09	2.76E-01	0.152	4.27E-02
UNC5H3	0.33	0.30	2.77E-01	0.154	4.03E-02
FGF 20	0.13	0.12	2.77E-01	-0.125	9.82E-02
PLPP	-0.09	0.08	2.78E-01	-0.074	3.25E-01
AMPK a1b1g1	-0.07	0.06	2.78E-01	-0.032	6.77E-01
CRDL1	-0.14	0.13	2.81E-01	-0.107	1.57E-01
ILT 4	-0.30	0.28	2.81E-01	0.021	7.78E-01
FGF23	0.30	0.28	2.82E-01	0.024	7.50E-01
NANOG	0.14	0.13	2.82E-01	-0.020	7.89E-01
MATN3	0.10	0.09	2.83E-01	-0.076	3.14E-01
PA2G4	-0.08	0.08	2.83E-01	-0.070	3.52E-01
RPS6KA3	-0.10	0.09	2.84E-01	-0.155	4.02E-02
MIF	-0.10	0.09	2.87E-01	0.084	2.70E-01
GDF2	0.10	0.09	2.87E-01	-0.061	4.18E-01
IL 7	0.11	0.11	2.87E-01	0.118	1.20E-01
Chitotriosidase 1	-0.12	0.12	2.87E-01	-0.105	1.89E-01
RUXF	-0.39	0.37	2.89E-01	0.253	6.77E-04
CTLA 4	0.15	0.14	2.90E-01	-0.001	9.92E-01
MOZ	0.12	0.11	2.91E-01	-0.050	5.07E-01
METAP1	-0.10	0.09	2.91E-01	-0.097	1.99E-01
Sphingosine kinase 1	-0.10	0.09	2.91E-01	0.074	3.28E-01
GSK 3 alpha beta	-0.10	0.09	2.91E-01	0.130	8.53E-02
LDH H 1	-0.09	0.08	2.92E-01	-0.003	9.70E-01
GOT1	-0.12	0.11	2.93E-01	-0.130	8.48E-02
TF	-0.15	0.14	2.93E-01	0.222	3.09E-03
RS7	-0.10	0.10	2.94E-01	-0.009	9.10E-01
ENA 78	0.09	0.08	2.95E-01	-0.151	4.48E-02
CAMK2A	-0.09	0.09	2.95E-01	-0.121	1.09E-01
Keratin 18	-0.12	0.11	2.96E-01	-0.129	8.90E-02
C3adesArg	0.16	0.15	2.96E-01	0.084	2.66E-01
CBPE	-0.13	0.13	2.97E-01	-0.073	3.34E-01
LSAMP	0.10	0.09	2.98E-01	-0.030	6.94E-01
LRRT1	0.09	0.09	2.98E-01	0.086	2.52E-01
HPV E7 Type18	0.10	0.10	2.98E-01	-0.053	4.85E-01
FER	-0.10	0.10	2.98E-01	-0.073	3.37E-01
CaMKK alpha	-0.09	0.09	2.99E-01	0.149	4.86E-02

IF4G2	-0.11	0.10	2.99E-01	-0.100	1.87E-01
PPIE	-0.09	0.09	2.99E-01	-0.077	3.08E-01
CDK8 cyclin C	-0.10	0.09	3.00E-01	-0.167	2.61E-02
GITR	-0.16	0.15	3.01E-01	-0.104	1.69E-01
JAM C	-0.09	0.09	3.01E-01	-0.115	1.27E-01
Karyopherin a2	0.11	0.11	3.03E-01	-0.086	2.55E-01
Sorting nexin 4	-0.10	0.10	3.03E-01	-0.079	2.98E-01
CRTAM	-0.18	0.18	3.04E-01	0.089	2.37E-01
Caspase 2	0.08	0.08	3.05E-01	-0.077	3.12E-01
PGCB	-0.10	0.10	3.06E-01	-0.312	2.33E-05
TSH	0.19	0.18	3.07E-01	0.075	3.19E-01
Laminin	0.25	0.25	3.08E-01	-0.080	2.92E-01
UB2L3	-0.08	0.08	3.09E-01	0.210	5.11E-03
Triosephosphate isomerase	-0.08	0.08	3.10E-01	0.089	2.37E-01
KI2L4	-0.09	0.09	3.11E-01	0.067	3.76E-01
DUS3	-0.09	0.09	3.11E-01	-0.045	5.53E-01
RBP	0.09	0.09	3.13E-01	-0.158	3.56E-02
EG VEGF	0.19	0.19	3.13E-01	0.016	8.38E-01
C5b 6 Complex	0.12	0.12	3.14E-01	0.188	1.24E-02
TSG 6	-0.10	0.10	3.14E-01	-0.013	8.59E-01
phosphoglycerate kinase 1	-0.11	0.11	3.14E-01	-0.141	6.14E-02
kallikrein 8	0.12	0.12	3.14E-01	-0.043	5.71E-01
Thrombin	0.10	0.10	3.15E-01	-0.044	5.59E-01
PKC A	-0.10	0.10	3.17E-01	-0.345	2.77E-06
TGF b2	-0.08	0.08	3.17E-01	-0.023	7.66E-01
PRL	-0.08	0.08	3.20E-01	-0.103	1.74E-01
Ephrin A4	0.21	0.21	3.21E-01	-0.141	6.18E-02
kallikrein 12	0.10	0.10	3.21E-01	-0.075	3.22E-01
TS	-0.14	0.14	3.23E-01	0.093	2.21E-01
SLAF7	-0.08	0.09	3.24E-01	-0.069	3.62E-01
Protein S	0.21	0.21	3.24E-01	0.076	3.12E-01
PDPK1	-0.10	0.10	3.25E-01	0.031	6.78E-01
a2 Macroglobulin	0.10	0.10	3.25E-01	-0.040	5.97E-01
IFN g	-0.09	0.09	3.26E-01	-0.043	5.71E-01
LEAP 1	0.12	0.12	3.26E-01	0.092	2.25E-01
CLC1B	-0.11	0.11	3.26E-01	-0.043	5.68E-01
IL 17 sR	0.25	0.25	3.26E-01	-0.090	2.36E-01
Endostatin	0.10	0.11	3.27E-01	0.072	3.40E-01
AREG	-0.08	0.08	3.27E-01	0.033	6.63E-01
Nucleoside diphosphate kinase A	-0.08	0.08	3.28E-01	0.167	2.74E-02
Midkine	-0.26	0.27	3.31E-01	-0.079	2.95E-01
PAPP A	0.24	0.25	3.32E-01	-0.060	4.25E-01
Peroxiredoxin 1	-0.07	0.07	3.33E-01	-0.104	1.70E-01
FGF9	0.11	0.12	3.33E-01	0.011	8.87E-01
Cystatin S	-0.11	0.11	3.33E-01	-0.002	9.81E-01

GP1BA	0.10	0.10	3.34E-01	-0.134	7.65E-02
Cadherin 2	0.08	0.09	3.35E-01	-0.074	3.27E-01
NRX1B	-0.22	0.22	3.36E-01	-0.077	3.07E-01
SMAC	0.11	0.11	3.39E-01	-0.169	2.44E-02
TGF b R III	-0.08	0.09	3.39E-01	-0.063	4.06E-01
Cytochrome P450 3A4	-0.09	0.09	3.41E-01	-0.133	7.95E-02
URB	-0.10	0.10	3.42E-01	-0.223	2.85E-03
PD L2	-0.13	0.14	3.42E-01	-0.021	7.83E-01
Rb	0.10	0.11	3.43E-01	-0.136	7.18E-02
DLRB1	-0.09	0.10	3.43E-01	-0.093	2.18E-01
Myeloperoxidase	0.13	0.13	3.44E-01	0.018	8.11E-01
JAG2	-0.09	0.09	3.45E-01	0.032	6.69E-01
PERL	-0.08	0.09	3.47E-01	0.036	6.35E-01
Contactin 5	0.11	0.12	3.48E-01	-0.104	1.70E-01
Glucocorticoid receptor	0.12	0.13	3.48E-01	-0.121	1.08E-01
PSMA	0.09	0.10	3.48E-01	-0.054	4.77E-01
Lysozyme	0.09	0.10	3.48E-01	-0.057	4.54E-01
ARSB	0.21	0.23	3.49E-01	0.114	1.32E-01
Cyclin B1	0.08	0.09	3.49E-01	-0.061	4.20E-01
SLPI	0.09	0.10	3.50E-01	-0.059	4.38E-01
IL 23	0.10	0.11	3.51E-01	-0.225	2.73E-03
Coagulation Factor IX	0.18	0.19	3.51E-01	-0.051	5.01E-01
PLXC1	0.42	0.45	3.52E-01	0.145	5.53E-02
SRCN1	-0.09	0.09	3.52E-01	0.149	4.78E-02
HSP 40	-0.08	0.09	3.52E-01	0.065	3.91E-01
PDE3A	-0.16	0.17	3.53E-01	-0.037	6.22E-01
XEDAR	-0.13	0.15	3.56E-01	0.119	1.13E-01
C1s	0.09	0.09	3.56E-01	0.008	9.20E-01
bFGF	-0.09	0.10	3.56E-01	-0.141	6.15E-02
HGFA	0.06	0.06	3.57E-01	-0.063	4.04E-01
Carbonic anhydrase VII	-0.07	0.08	3.57E-01	0.015	8.43E-01
M2 PK	-0.08	0.09	3.58E-01	-0.100	1.85E-01
PDK1	0.18	0.20	3.58E-01	-0.137	6.90E-02
CXCL16 soluble	0.31	0.33	3.58E-01	-0.076	3.14E-01
SLAF5	-0.10	0.11	3.59E-01	0.128	8.99E-02
RAC1	-0.08	0.08	3.59E-01	0.118	1.17E-01
PSA1	-0.19	0.21	3.59E-01	-0.169	2.50E-02
PKC Z	-0.10	0.11	3.62E-01	-0.064	4.00E-01
IL22RA1	-0.08	0.09	3.63E-01	0.165	2.90E-02
Peroxiredoxin 6	-0.07	0.08	3.63E-01	0.052	4.88E-01
Spondin 1	-0.09	0.10	3.63E-01	0.011	8.86E-01
IL 2 sRg	-0.08	0.09	3.63E-01	0.082	2.79E-01
JAM B	0.43	0.48	3.64E-01	-0.006	9.37E-01
CATE	0.08	0.09	3.66E-01	-0.099	1.90E-01
ATS1	-0.09	0.10	3.66E-01	0.024	7.50E-01

PDGF AA	-0.09	0.09	3.67E-01	-0.144	5.52E-02
PTHrP	0.23	0.25	3.68E-01	0.159	3.47E-02
SP D	-0.07	0.08	3.69E-01	0.149	4.88E-02
LIF sR	-0.10	0.11	3.69E-01	-0.017	8.20E-01
PSA6	-0.08	0.09	3.71E-01	0.021	7.85E-01
BSP	-0.22	0.24	3.71E-01	0.127	9.38E-02
PBEF	-0.11	0.12	3.72E-01	-0.094	2.11E-01
MMP 7	-0.24	0.27	3.74E-01	-0.067	3.77E-01
WNT7A	-0.23	0.26	3.76E-01	-0.061	4.18E-01
Coactosin like protein	0.26	0.29	3.76E-01	-0.007	9.24E-01
IL 17B R	-0.13	0.15	3.78E-01	-0.167	2.65E-02
SREC II	-0.08	0.09	3.79E-01	0.119	1.18E-01
KPCI	-0.07	0.08	3.80E-01	-0.004	9.57E-01
ROBO2	0.09	0.11	3.81E-01	-0.065	3.87E-01
DnaJ homolog	0.19	0.22	3.81E-01	0.119	1.16E-01
Myoglobin	0.07	0.08	3.82E-01	-0.013	8.62E-01
ON	-0.08	0.09	3.84E-01	-0.196	9.20E-03
ACTH	-0.05	0.06	3.84E-01	0.186	1.34E-02
Transketolase	-0.07	0.08	3.86E-01	0.074	3.27E-01
LRRT3	-0.15	0.17	3.86E-01	-0.042	5.83E-01
HPG	-0.09	0.11	3.87E-01	-0.087	2.49E-01
EMR2	0.10	0.11	3.87E-01	-0.116	1.23E-01
C3	0.15	0.17	3.87E-01	0.018	8.10E-01
IL 17 RD	0.07	0.08	3.87E-01	0.105	1.67E-01
Cathepsin S	0.28	0.33	3.87E-01	0.051	5.02E-01
Cathepsin A	-0.07	0.08	3.89E-01	0.091	2.29E-01
HTRA2	-0.11	0.13	3.90E-01	0.138	6.69E-02
annexin I	0.15	0.17	3.90E-01	0.096	2.03E-01
GNS	0.11	0.13	3.91E-01	-0.001	9.84E-01
IL 17F	0.09	0.10	3.92E-01	-0.031	6.84E-01
Fibronectin	-0.12	0.14	3.92E-01	-0.154	4.10E-02
FGF 4	0.08	0.10	3.93E-01	0.000	9.97E-01
Gro a	-0.09	0.11	3.94E-01	-0.064	3.95E-01
CONA1	-0.10	0.11	3.95E-01	0.035	6.44E-01
MCP 1	-0.15	0.18	3.99E-01	0.077	3.07E-01
LIGHT	0.08	0.10	4.00E-01	-0.156	3.85E-02
X6 Phosphogluconate dehydrogenase	-0.07	0.08	4.00E-01	0.002	9.78E-01
IFN lambda 1	-0.08	0.10	4.01E-01	-0.034	6.51E-01
Aurora kinase A	-0.06	0.07	4.01E-01	0.241	1.27E-03
MP2K2	0.20	0.24	4.02E-01	0.170	2.41E-02
ANGL3	-0.07	0.09	4.02E-01	0.038	6.18E-01
UBE2N	-0.06	0.07	4.02E-01	-0.032	6.77E-01
Coagulation Factor Xa	0.16	0.19	4.03E-01	-0.037	6.30E-01
DYRK3	0.09	0.11	4.03E-01	-0.018	8.15E-01
PDGF BB	-0.08	0.10	4.04E-01	0.063	4.08E-01

OMD	0.18	0.22	4.04E-01	-0.057	4.51E-01
CHK1	0.08	0.10	4.06E-01	-0.013	8.65E-01
TFF3	-0.09	0.11	4.06E-01	0.057	4.54E-01
DERM	0.09	0.10	4.06E-01	-0.031	6.85E-01
Ubiquitin 1	-0.05	0.07	4.10E-01	0.020	7.94E-01
Proteinase 3	0.10	0.12	4.11E-01	-0.051	4.99E-01
EMAP 2	0.09	0.10	4.11E-01	0.118	1.20E-01
B7	0.10	0.13	4.14E-01	0.035	6.39E-01
CHST2	-0.10	0.12	4.15E-01	0.111	1.42E-01
PGRP S	-0.07	0.09	4.17E-01	-0.048	5.22E-01
MRC2	-0.09	0.11	4.18E-01	0.098	1.94E-01
H6ST1	0.09	0.11	4.19E-01	-0.108	1.54E-01
Granzyme H	-0.10	0.12	4.19E-01	-0.003	9.67E-01
vWF	0.20	0.25	4.21E-01	-0.034	6.51E-01
IL 19	-0.08	0.10	4.21E-01	0.074	3.27E-01
STAB2	0.09	0.11	4.21E-01	-0.116	1.23E-01
WISP 1	0.08	0.10	4.22E-01	0.084	2.70E-01
ARTS1	0.08	0.10	4.22E-01	0.089	2.39E-01
UFM1	-0.08	0.10	4.23E-01	-0.026	7.31E-01
Cathepsin V	0.10	0.12	4.23E-01	0.072	3.44E-01
Arylsulfatase A	-0.08	0.10	4.24E-01	0.013	8.64E-01
COMMD7	-0.08	0.10	4.24E-01	0.059	4.38E-01
KPCT	-0.07	0.09	4.27E-01	-0.073	3.36E-01
CATZ	0.09	0.11	4.28E-01	-0.114	1.32E-01
C4b	0.07	0.08	4.29E-01	-0.056	4.56E-01
C1QBP	0.08	0.11	4.29E-01	-0.032	6.73E-01
GAS1	0.09	0.11	4.29E-01	-0.027	7.20E-01
FCGR1	-0.07	0.09	4.32E-01	0.053	4.85E-01
pTEN	-0.09	0.11	4.32E-01	0.099	1.90E-01
FGF 8A	0.09	0.11	4.36E-01	-0.053	4.87E-01
RSPO2	-0.09	0.11	4.37E-01	0.086	2.55E-01
BPI	0.08	0.10	4.37E-01	0.036	6.36E-01
Vasoactive Intestinal Peptide	-0.07	0.09	4.39E-01	0.123	1.05E-01
BARK1	-0.08	0.10	4.39E-01	0.031	6.81E-01
BDNF	-0.07	0.09	4.39E-01	0.208	5.50E-03
Testican 1	-0.07	0.09	4.42E-01	0.054	4.77E-01
Cystatin C	0.31	0.40	4.42E-01	0.054	4.82E-01
Semaphorin 6A	0.10	0.13	4.44E-01	0.108	1.53E-01
ITI heavy chain H4	0.07	0.10	4.45E-01	0.218	3.58E-03
PECAM 1	0.08	0.11	4.45E-01	-0.067	3.77E-01
Carbonic anhydrase XIII	-0.07	0.09	4.45E-01	0.175	2.02E-02
PAK6	-0.07	0.09	4.46E-01	-0.146	5.42E-02
CD30 Ligand	0.32	0.41	4.46E-01	-0.063	4.03E-01
SHP 2	-0.07	0.10	4.48E-01	0.096	2.05E-01
MBL	-0.05	0.07	4.50E-01	-0.005	9.51E-01

EPHB2	-0.07	0.09	4.50E-01	0.007	9.25E-01
PACAP 38	0.10	0.13	4.50E-01	0.120	1.13E-01
NLGNX	-0.19	0.25	4.50E-01	-0.239	1.38E-03
TBP	0.07	0.09	4.51E-01	0.194	1.03E-02
AMNLS	-0.08	0.10	4.53E-01	0.035	6.45E-01
IL 1 sR9	0.07	0.09	4.53E-01	-0.107	1.57E-01
PTK6	-0.10	0.14	4.53E-01	-0.029	6.99E-01
EPHAA	0.07	0.10	4.53E-01	-0.085	2.63E-01
Fractalkine CX3CL 1	0.16	0.21	4.53E-01	-0.036	6.31E-01
VCAM 1	0.36	0.48	4.55E-01	-0.125	9.68E-02
IL 6	-0.07	0.09	4.57E-01	0.060	4.29E-01
Coagulation Factor IXab	0.13	0.18	4.58E-01	0.062	4.13E-01
KREM2	-0.07	0.10	4.59E-01	-0.016	8.36E-01
MMP 16	-0.08	0.10	4.59E-01	-0.165	2.90E-02
Galectin 8	0.07	0.10	4.59E-01	0.058	4.44E-01
Fas ligand soluble	0.09	0.12	4.59E-01	-0.155	3.95E-02
Cripto	0.07	0.10	4.62E-01	0.148	5.05E-02
Angiopoietin 4	-0.08	0.11	4.64E-01	0.155	3.93E-02
IGF II receptor	0.07	0.09	4.64E-01	-0.013	8.68E-01
RANTES	-0.08	0.11	4.64E-01	-0.028	7.12E-01
ACE2	0.06	0.08	4.65E-01	0.079	2.95E-01
SEPR	-0.09	0.12	4.65E-01	-0.019	8.00E-01
UNC5H4	0.08	0.12	4.68E-01	0.180	1.66E-02
ESAM	-0.09	0.13	4.69E-01	0.156	3.92E-02
Sialoadhesin	0.14	0.19	4.70E-01	-0.006	9.37E-01
FGF7	-0.07	0.10	4.71E-01	-0.056	4.61E-01
SPHK2	-0.06	0.09	4.74E-01	0.106	1.62E-01
MATK	-0.07	0.10	4.76E-01	-0.102	1.79E-01
NADPH P450 Oxidoreductase	0.15	0.21	4.79E-01	0.109	1.48E-01
SARP 2	0.22	0.31	4.80E-01	0.113	1.35E-01
IL 16	0.06	0.09	4.82E-01	0.048	5.22E-01
eIF 5A 1	-0.07	0.09	4.83E-01	-0.026	7.34E-01
KYNU	0.16	0.22	4.83E-01	0.084	2.65E-01
C34 gp41 HIV Fragment	-0.07	0.09	4.84E-01	-0.007	9.30E-01
sICAM 2	0.06	0.09	4.86E-01	-0.210	5.27E-03
CD48	0.33	0.48	4.86E-01	0.138	6.71E-02
MDHC	-0.05	0.07	4.87E-01	-0.053	4.83E-01
Ephrin B3	-0.08	0.11	4.90E-01	-0.080	2.90E-01
MIP 3b	0.18	0.27	4.94E-01	-0.167	2.63E-02
Tenascin	-0.07	0.11	4.95E-01	-0.133	8.00E-02
CDK5 p35	0.06	0.09	4.95E-01	0.037	6.24E-01
PUR8	-0.24	0.35	4.97E-01	0.017	8.23E-01
HNRPQ	-0.08	0.11	4.98E-01	-0.094	2.13E-01
PDGF Rb	0.09	0.13	5.00E-01	-0.009	9.02E-01
BCMA	-0.07	0.10	5.00E-01	-0.058	4.41E-01

PDGF CC	0.06	0.09	5.01E-01	-0.049	5.19E-01
CHL1	0.07	0.11	5.01E-01	0.049	5.16E-01
PAI 1	-0.06	0.09	5.02E-01	0.002	9.81E-01
CAMK1	0.06	0.09	5.02E-01	-0.007	9.22E-01
IL 4	-0.16	0.23	5.02E-01	-0.100	1.87E-01
TARC	-0.06	0.09	5.02E-01	-0.182	1.58E-02
Eotaxin	-0.06	0.09	5.03E-01	0.028	7.12E-01
CK2 A1 B	-0.06	0.09	5.04E-01	-0.014	8.55E-01
NKp46	-0.09	0.13	5.05E-01	0.195	9.12E-03
TRAIL R1	0.07	0.10	5.06E-01	-0.084	2.69E-01
Azurocidin	-0.06	0.09	5.06E-01	0.098	1.96E-01
Cyclophilin A	-0.05	0.08	5.07E-01	-0.047	5.38E-01
SSRP1	-0.07	0.11	5.07E-01	0.121	1.08E-01
UFC1	-0.07	0.11	5.08E-01	0.044	5.60E-01
FST	-0.06	0.10	5.10E-01	0.070	3.57E-01
Luteinizing hormone	0.05	0.07	5.10E-01	-0.041	5.93E-01
B7 H1	-0.13	0.19	5.11E-01	-0.040	6.01E-01
RGM C	0.07	0.10	5.11E-01	-0.011	8.95E-01
Calpain I	-0.05	0.08	5.15E-01	-0.063	4.03E-01
BST1	-0.05	0.08	5.16E-01	0.152	4.36E-02
PAK7	0.09	0.14	5.16E-01	0.027	7.20E-01
YES	-0.07	0.10	5.17E-01	-0.066	3.82E-01
NUDC3	0.09	0.14	5.18E-01	-0.093	2.20E-01
LCK	0.20	0.30	5.18E-01	0.028	7.07E-01
TSP2	-0.16	0.25	5.18E-01	0.006	9.41E-01
Cofilin 1	-0.06	0.09	5.20E-01	0.067	3.75E-01
IL 18 Ra	0.13	0.20	5.20E-01	0.052	4.90E-01
DKK3	0.07	0.10	5.21E-01	0.012	8.78E-01
Elafin	0.06	0.10	5.22E-01	0.002	9.77E-01
hnRNP A B	-0.05	0.08	5.23E-01	0.099	1.93E-01
Semaphorin 3E	0.11	0.17	5.24E-01	-0.059	4.33E-01
CTGF	-0.15	0.24	5.24E-01	-0.226	2.54E-03
AIF1	0.05	0.09	5.24E-01	-0.174	2.03E-02
LCMT1	-0.06	0.10	5.26E-01	-0.082	2.78E-01
sICAM 5	0.08	0.12	5.27E-01	-0.018	8.17E-01
TGF b1	-0.07	0.11	5.27E-01	0.057	4.50E-01
MO2R1	-0.07	0.11	5.28E-01	-0.071	3.52E-01
CRK	-0.08	0.12	5.29E-01	0.049	5.18E-01
C2	-0.11	0.18	5.30E-01	0.072	3.39E-01
IgD	0.06	0.09	5.31E-01	0.073	3.39E-01
hnRNP A2 B1	-0.05	0.08	5.32E-01	-0.018	8.17E-01
HVEM	0.09	0.14	5.32E-01	0.128	9.08E-02
Lactoferrin	0.07	0.12	5.34E-01	-0.061	4.21E-01
TWEAKR	-0.13	0.21	5.35E-01	-0.045	5.56E-01
DMP1	-0.07	0.12	5.36E-01	-0.109	1.49E-01

ULBP 2	0.08	0.13	5.37E-01	-0.080	2.90E-01
CO8A1	0.09	0.15	5.37E-01	0.017	8.21E-01
HGF	-0.09	0.15	5.38E-01	-0.180	1.66E-02
ARMEL	0.18	0.29	5.38E-01	0.065	3.87E-01
GAPDH liver	-0.04	0.07	5.39E-01	0.134	7.69E-02
b NGF	-0.07	0.11	5.39E-01	0.045	5.51E-01
Thyroglobulin	0.06	0.11	5.42E-01	-0.059	4.38E-01
SIG14	-0.08	0.12	5.45E-01	0.005	9.44E-01
XPNPEP1	-0.07	0.12	5.45E-01	0.012	8.77E-01
IGFBP 1	-0.06	0.09	5.46E-01	0.063	4.07E-01
GSTA3	0.09	0.15	5.46E-01	0.147	5.04E-02
MMEL2	-0.07	0.11	5.46E-01	-0.109	1.50E-01
Nectin like protein 1	0.06	0.10	5.47E-01	0.059	4.36E-01
MMP 13	-0.06	0.10	5.48E-01	0.049	5.21E-01
IDS	0.09	0.16	5.49E-01	-0.077	3.08E-01
STX1a	0.06	0.09	5.49E-01	-0.013	8.68E-01
GDF 11	0.07	0.11	5.49E-01	0.036	6.37E-01
STRATIFIN	-0.05	0.09	5.52E-01	0.121	1.08E-01
Flt 3	-0.07	0.11	5.52E-01	0.021	7.83E-01
MRCKB	-0.06	0.11	5.53E-01	0.100	1.88E-01
Epo	0.12	0.20	5.54E-01	-0.016	8.29E-01
IL 7 Ra	-0.06	0.11	5.56E-01	-0.095	2.08E-01
Alkaline phosphatase bone	0.06	0.11	5.61E-01	0.032	6.69E-01
SGTA	-0.05	0.09	5.63E-01	-0.026	7.35E-01
BCL2 like 1 protein	-0.06	0.11	5.64E-01	-0.106	1.60E-01
ENPP7	-0.12	0.21	5.66E-01	0.124	9.93E-02
BMP RII	-0.13	0.22	5.66E-01	-0.053	4.80E-01
Cadherin 12	-0.05	0.09	5.67E-01	-0.059	4.33E-01
MK13	-0.04	0.08	5.68E-01	-0.084	2.66E-01
SCF sR	0.06	0.11	5.68E-01	-0.022	7.69E-01
SIRT2	-0.05	0.09	5.69E-01	0.010	9.00E-01
FGFR 2	-0.06	0.10	5.71E-01	0.220	3.26E-03
SOD	-0.05	0.09	5.72E-01	-0.037	6.22E-01
NSF1C	-0.05	0.09	5.73E-01	-0.084	2.65E-01
MDM2	0.08	0.14	5.74E-01	0.034	6.54E-01
Prolactin Receptor	-0.17	0.31	5.76E-01	0.043	5.72E-01
FLRT1	-0.06	0.10	5.76E-01	-0.019	8.02E-01
GCKR	-0.05	0.09	5.76E-01	-0.138	6.78E-02
Plasmin	-0.17	0.31	5.77E-01	-0.048	5.23E-01
FAK1	0.07	0.13	5.86E-01	0.035	6.47E-01
Chymase	-0.05	0.09	5.87E-01	0.064	3.97E-01
ST4S6	-0.16	0.30	5.88E-01	0.153	4.24E-02
PPIB	-0.06	0.11	5.88E-01	-0.026	7.29E-01
Gro b g	-0.05	0.09	5.88E-01	0.204	6.45E-03
Somatostatin 28	0.06	0.10	5.88E-01	-0.058	4.41E-01

TNF sR I	0.25	0.46	5.90E-01	-0.194	9.71E-03
EphB6	0.06	0.12	5.90E-01	-0.080	2.90E-01
Rab GDP dissociation inhibitor beta	-0.04	0.07	5.95E-01	-0.034	6.56E-01
DEAD box protein 19B	0.05	0.09	5.97E-01	-0.081	2.86E-01
FGF 5	-0.07	0.14	5.98E-01	0.031	6.81E-01
SHBG	-0.06	0.10	5.98E-01	-0.027	7.24E-01
DRAK2	-0.05	0.09	5.98E-01	0.025	7.37E-01
TGF b3	0.05	0.10	5.99E-01	-0.117	1.22E-01
Noggin	0.06	0.12	5.99E-01	-0.084	2.67E-01
PLK 1	0.10	0.18	5.99E-01	0.149	4.75E-02
Protease nexin I	-0.09	0.16	6.01E-01	0.078	3.05E-01
IDE	-0.05	0.09	6.01E-01	0.033	6.59E-01
NAGK	-0.04	0.08	6.02E-01	0.037	6.21E-01
Cadherin 6	0.05	0.10	6.03E-01	-0.127	9.30E-02
I TAC	-0.09	0.17	6.03E-01	-0.103	1.71E-01
sCD4	0.06	0.12	6.03E-01	-0.005	9.52E-01
CTACK	0.05	0.10	6.06E-01	0.009	9.04E-01
LGMN	-0.04	0.08	6.08E-01	-0.148	4.97E-02
EDAR	-0.04	0.08	6.09E-01	-0.039	6.05E-01
Cathepsin H	-0.07	0.14	6.09E-01	0.133	7.86E-02
Integrin aVb5	-0.04	0.07	6.10E-01	0.003	9.64E-01
TPSG1	0.05	0.11	6.10E-01	-0.066	3.83E-01
BMPR1A	0.05	0.10	6.10E-01	0.035	6.44E-01
transcription factor MLR1 isoform CRA_b	-0.13	0.25	6.10E-01	0.061	4.22E-01
a1 Antichymotrypsin	0.06	0.12	6.12E-01	0.148	5.00E-02
IL 17 RC	-0.05	0.10	6.12E-01	0.053	4.87E-01
IGFBP 6	-0.07	0.14	6.14E-01	-0.028	7.11E-01
FGF 19	-0.04	0.09	6.20E-01	-0.058	4.45E-01
NKp30	0.16	0.33	6.22E-01	0.014	8.49E-01
CKAP2	0.05	0.10	6.22E-01	0.000	9.97E-01
COX 2	-0.05	0.11	6.22E-01	-0.028	7.11E-01
RAN	-0.04	0.08	6.24E-01	-0.145	5.51E-02
Nectin like protein 2	0.06	0.12	6.24E-01	-0.049	5.20E-01
ROBO3	-0.06	0.12	6.24E-01	0.122	1.07E-01
MAPK5	-0.09	0.19	6.26E-01	-0.053	4.82E-01
Macrophage mannose receptor	0.13	0.26	6.28E-01	-0.022	7.68E-01
PARC	-0.14	0.28	6.29E-01	0.164	2.95E-02
Elastase	0.06	0.13	6.30E-01	0.051	5.04E-01
LKHA4	0.06	0.13	6.30E-01	-0.017	8.19E-01
TFPI	0.18	0.37	6.30E-01	-0.052	4.93E-01
Glutathione S transferase Pi	-0.06	0.12	6.32E-01	-0.019	7.99E-01
IL 22	0.08	0.17	6.35E-01	0.011	8.87E-01
DSC3	0.05	0.10	6.36E-01	-0.042	5.76E-01
TIMP 3	-0.05	0.10	6.37E-01	-0.061	4.23E-01
Epithelial cell kinase	0.10	0.22	6.40E-01	0.144	6.87E-02

MSP	0.08	0.17	6.46E-01	0.070	3.55E-01
B7 H2	-0.05	0.10	6.47E-01	-0.079	2.95E-01
ERBB2	-0.08	0.18	6.49E-01	0.202	7.09E-03
PK3CG	-0.15	0.33	6.50E-01	-0.088	2.45E-01
TAJ	-0.10	0.22	6.51E-01	0.173	2.11E-02
NDP kinase B	-0.05	0.10	6.53E-01	-0.015	8.47E-01
IL 1b	-0.08	0.17	6.54E-01	0.076	3.15E-01
IL 5 Ra	-0.09	0.20	6.55E-01	-0.073	3.33E-01
X40S ribosomal protein SA	-0.04	0.09	6.55E-01	0.150	4.61E-02
G CSF R	-0.09	0.20	6.55E-01	0.110	1.45E-01
BMP10	0.06	0.14	6.57E-01	0.027	7.17E-01
COLEC12	0.05	0.10	6.59E-01	-0.020	7.91E-01
SPARCL1	0.04	0.09	6.59E-01	-0.080	2.91E-01
ABL1	-0.06	0.13	6.60E-01	0.029	7.05E-01
CSK21	-0.04	0.10	6.67E-01	0.117	1.21E-01
complement factor H related 5	0.05	0.11	6.67E-01	0.106	1.63E-01
Carbonic anhydrase I	0.04	0.10	6.69E-01	-0.043	5.66E-01
Glutamate carboxypeptidase	-0.05	0.11	6.70E-01	0.032	6.72E-01
iC3b	-0.04	0.10	6.71E-01	-0.014	8.49E-01
EDA	0.11	0.25	6.72E-01	0.064	3.95E-01
HMG 1	-0.04	0.09	6.73E-01	-0.090	2.37E-01
RGMA	0.04	0.09	6.73E-01	-0.028	7.11E-01
TrkA	-0.03	0.08	6.75E-01	0.062	4.18E-01
SLIK5	0.05	0.12	6.77E-01	-0.021	7.84E-01
ADAMTS 5	0.04	0.10	6.80E-01	0.035	6.41E-01
PRKACA	-0.05	0.11	6.81E-01	0.083	2.71E-01
Histone H1 2	-0.04	0.10	6.81E-01	-0.060	4.32E-01
Myokinase human	-0.03	0.06	6.81E-01	0.004	9.57E-01
IL 18 BPa	0.11	0.26	6.82E-01	-0.077	3.10E-01
CD23	-0.06	0.14	6.88E-01	0.008	9.13E-01
contactin 1	-0.04	0.10	6.89E-01	-0.103	1.72E-01
SET	0.05	0.14	6.91E-01	-0.014	8.56E-01
PACAP 27	0.04	0.11	6.91E-01	-0.030	6.88E-01
Caspase 10	-0.04	0.11	6.91E-01	-0.091	2.53E-01
CAPG	-0.04	0.10	6.92E-01	0.016	8.37E-01
CAD15	0.04	0.10	6.93E-01	-0.020	7.91E-01
TCPTP	0.10	0.26	6.93E-01	-0.119	1.13E-01
TACI	0.04	0.09	6.93E-01	-0.023	7.57E-01
Artemin	-0.12	0.30	6.96E-01	-0.008	9.16E-01
PIK3CA PIK3R1	-0.04	0.10	6.97E-01	-0.006	9.38E-01
OSM	0.10	0.25	6.97E-01	-0.077	3.13E-01
JAML1	0.03	0.08	6.97E-01	0.020	7.94E-01
C4	0.09	0.24	6.98E-01	0.005	9.52E-01
PDE4D	0.04	0.11	6.99E-01	0.127	9.23E-02
PSD7	0.04	0.10	6.99E-01	-0.018	8.19E-01

Insulin	0.03	0.09	7.00E-01	-0.004	9.60E-01
IL 20 Ra	-0.04	0.10	7.04E-01	-0.145	5.38E-02
PSME1	-0.03	0.08	7.05E-01	0.051	5.02E-01
OCAD1	-0.03	0.09	7.07E-01	0.059	4.39E-01
GFRa 1	0.08	0.22	7.13E-01	0.112	1.39E-01
C5a	0.04	0.11	7.13E-01	-0.144	5.58E-02
Hemopexin	0.04	0.11	7.16E-01	-0.045	5.48E-01
resistin	-0.05	0.13	7.16E-01	0.043	5.66E-01
Clusterin	0.03	0.08	7.17E-01	0.016	8.38E-01
MIP 5	0.03	0.10	7.18E-01	0.012	8.78E-01
TSLP	-0.04	0.10	7.19E-01	0.133	7.91E-02
RGMB	0.04	0.11	7.19E-01	0.055	4.65E-01
BOC	-0.04	0.10	7.22E-01	-0.135	7.36E-02
CBG	-0.03	0.08	7.24E-01	0.112	1.38E-01
NPS PLA2	0.06	0.17	7.26E-01	0.023	7.57E-01
IL 1Rrp2	0.03	0.10	7.26E-01	0.144	5.64E-02
NOTC2	0.03	0.10	7.26E-01	0.056	4.60E-01
HDAC8	-0.03	0.10	7.26E-01	-0.032	6.72E-01
DRR1	-0.11	0.31	7.27E-01	0.169	2.41E-02
MEPE	0.04	0.13	7.27E-01	0.002	9.76E-01
IL 11	-0.06	0.16	7.28E-01	-0.123	1.04E-01
EphB4	-0.03	0.08	7.30E-01	-0.037	6.30E-01
Topoisomerase I	-0.04	0.11	7.30E-01	0.029	6.99E-01
hnRNP K	-0.04	0.12	7.31E-01	0.159	3.54E-02
Hemoglobin	0.08	0.24	7.33E-01	-0.038	6.19E-01
Granzyme B	-0.04	0.10	7.35E-01	0.019	8.03E-01
IL 13 Ra1	0.03	0.09	7.39E-01	-0.030	6.89E-01
Macrophage scavenger receptor	-0.04	0.11	7.39E-01	0.113	1.34E-01
PTN	-0.11	0.32	7.39E-01	-0.045	5.51E-01
C5	0.04	0.11	7.41E-01	0.011	8.84E-01
IMDH1	-0.03	0.09	7.43E-01	0.039	6.02E-01
Phosphoglycerate mutase 1	0.04	0.11	7.43E-01	0.044	5.59E-01
IL 17D	-0.03	0.10	7.45E-01	-0.138	6.68E-02
FGF 12	0.03	0.10	7.45E-01	0.055	4.67E-01
Factor D	-0.04	0.13	7.45E-01	0.017	8.27E-01
MEK1	-0.04	0.13	7.46E-01	-0.071	3.49E-01
Bcl 2	-0.03	0.09	7.47E-01	0.084	2.66E-01
DcR3	-0.03	0.10	7.49E-01	0.104	1.68E-01
FABPE	0.05	0.15	7.50E-01	0.023	7.66E-01
Ck b 8 1	0.02	0.08	7.50E-01	-0.064	4.01E-01
LBP	-0.03	0.10	7.52E-01	0.076	3.16E-01
FGF 18	0.08	0.25	7.52E-01	-0.133	7.77E-02
Gelsolin	-0.03	0.10	7.53E-01	-0.092	2.23E-01
IL 12 RB2	0.05	0.17	7.53E-01	0.081	2.85E-01
Cathepsin G	0.03	0.09	7.55E-01	0.007	9.31E-01

Protein disulfide isomerase	-0.03	0.09	7.60E-01	0.044	5.59E-01
LIN7B	0.03	0.11	7.60E-01	-0.136	7.22E-02
ERBB4	0.06	0.21	7.60E-01	-0.031	6.81E-01
BRF 1	0.03	0.09	7.62E-01	0.177	1.86E-02
ENTP3	-0.09	0.31	7.62E-01	0.100	1.86E-01
MAPKAPK3	-0.04	0.12	7.67E-01	0.128	9.08E-02
IGFBP 7	-0.12	0.42	7.68E-01	-0.014	8.57E-01
CDC37	-0.03	0.09	7.69E-01	0.017	8.25E-01
HAI 1	0.03	0.10	7.69E-01	0.147	5.23E-02
Peroxiredoxin 5	0.03	0.10	7.71E-01	0.041	5.92E-01
VEGF	-0.02	0.09	7.71E-01	-0.097	2.02E-01
IL 2 sRa	-0.05	0.18	7.72E-01	0.074	3.27E-01
BMP 6	-0.03	0.11	7.72E-01	-0.012	8.77E-01
MED 1	0.03	0.11	7.76E-01	-0.039	6.04E-01
CFC1	-0.03	0.10	7.76E-01	-0.076	3.14E-01
GRN	-0.14	0.51	7.77E-01	-0.096	2.05E-01
Ku70	0.03	0.10	7.81E-01	-0.002	9.79E-01
NovH	-0.07	0.25	7.81E-01	0.050	5.14E-01
Kallikrein 4	-0.01	0.04	7.82E-01	0.038	6.11E-01
IMB1	-0.03	0.11	7.83E-01	0.105	1.63E-01
GP114	0.03	0.11	7.84E-01	-0.177	1.82E-02
Siglec 9	0.03	0.11	7.85E-01	-0.077	3.07E-01
GIIE	0.03	0.10	7.87E-01	0.084	2.71E-01
IL 5	0.03	0.12	7.89E-01	-0.016	8.33E-01
MICA	0.03	0.11	7.89E-01	0.058	4.41E-01
C1 Esterase Inhibitor	-0.03	0.11	7.91E-01	0.196	8.87E-03
IgM	-0.08	0.29	7.92E-01	-0.048	5.29E-01
NKG2D	-0.05	0.18	7.93E-01	-0.016	8.29E-01
FGFR 3	0.02	0.07	7.97E-01	-0.058	4.42E-01
Contactin 4	0.03	0.10	7.97E-01	-0.059	4.40E-01
IL 8	0.06	0.23	7.97E-01	0.095	2.12E-01
PDE9A	0.10	0.37	7.97E-01	0.016	8.30E-01
PARK7	0.06	0.22	7.98E-01	-0.013	8.68E-01
Renin	0.06	0.24	7.99E-01	-0.111	1.40E-01
MCP 4	-0.02	0.09	8.00E-01	0.026	7.29E-01
TWEAK	0.02	0.09	8.03E-01	0.017	8.24E-01
Siglec 7	-0.03	0.11	8.06E-01	0.022	7.75E-01
LRP8	0.03	0.10	8.08E-01	0.008	9.19E-01
MMP 17	0.02	0.09	8.08E-01	0.052	4.93E-01
C3b	0.04	0.16	8.09E-01	-0.092	2.24E-01
NG36	-0.03	0.11	8.09E-01	0.058	4.47E-01
CLC7A	0.03	0.12	8.11E-01	0.078	3.05E-01
TYK2	0.02	0.07	8.12E-01	-0.056	4.58E-01
MBD4	-0.02	0.08	8.12E-01	0.023	7.60E-01
BGN	0.04	0.15	8.13E-01	-0.022	7.72E-01

AN32B	0.02	0.07	8.14E-01	-0.007	9.23E-01
cIAP 2	0.03	0.12	8.14E-01	-0.005	9.44E-01
Cathepsin D	-0.03	0.11	8.20E-01	-0.020	7.95E-01
BCAM	0.06	0.26	8.22E-01	0.020	7.89E-01
CK2 A2 B	-0.02	0.10	8.23E-01	0.020	7.94E-01
RBM39	-0.02	0.10	8.24E-01	-0.003	9.68E-01
DAF	-0.02	0.10	8.24E-01	0.066	3.84E-01
IFN lambda 2	-0.02	0.07	8.25E-01	-0.156	3.90E-02
NCK1	-0.04	0.20	8.28E-01	0.184	1.44E-02
PPAC	0.02	0.09	8.28E-01	-0.013	8.65E-01
IL 6 sRa	-0.02	0.09	8.28E-01	-0.031	6.86E-01
HB EGF	-0.02	0.10	8.29E-01	0.150	4.61E-02
GFRa 3	-0.02	0.11	8.30E-01	0.085	2.58E-01
CDK1 cyclin B	-0.02	0.10	8.31E-01	-0.074	3.30E-01
LAG 1	0.02	0.11	8.32E-01	0.062	4.10E-01
PTH	-0.03	0.12	8.33E-01	0.037	6.25E-01
Coagulation Factor X	-0.11	0.53	8.34E-01	-0.068	3.70E-01
cGMP stimulated PDE	0.02	0.08	8.35E-01	0.004	9.56E-01
C1q	0.03	0.14	8.37E-01	-0.003	9.71E-01
AGR2	0.03	0.15	8.39E-01	0.083	2.75E-01
MMP 10	0.02	0.12	8.39E-01	0.069	3.66E-01
IF4A3	0.02	0.10	8.44E-01	0.091	2.28E-01
Nr CAM	0.04	0.22	8.45E-01	-0.004	9.55E-01
IL 17	0.01	0.07	8.48E-01	0.028	7.14E-01
TECK	-0.02	0.09	8.48E-01	-0.019	7.98E-01
OX40 Ligand	0.02	0.13	8.51E-01	-0.141	6.24E-02
EPO R	-0.02	0.11	8.52E-01	0.088	2.43E-01
LD78 beta	0.02	0.11	8.53E-01	0.075	3.20E-01
TNR4	-0.01	0.07	8.54E-01	0.015	8.43E-01
SORC2	-0.02	0.11	8.55E-01	0.114	1.35E-01
GPC5	0.03	0.16	8.56E-01	0.016	8.36E-01
MMP 2	-0.02	0.10	8.59E-01	-0.012	8.77E-01
CHST6	0.03	0.14	8.61E-01	0.139	6.47E-02
Layilin	0.02	0.11	8.61E-01	-0.007	9.28E-01
Cytidylate kinase	0.02	0.11	8.61E-01	0.039	6.06E-01
IL 1 R4	0.03	0.20	8.64E-01	-0.029	7.04E-01
IGFBP 5	0.02	0.12	8.65E-01	-0.081	2.86E-01
ROR1	0.02	0.10	8.66E-01	0.017	8.19E-01
GPC2	0.02	0.12	8.72E-01	-0.006	9.35E-01
Mammaglobin 2	-0.05	0.28	8.73E-01	-0.075	3.21E-01
ASM3A	0.01	0.09	8.74E-01	0.106	1.63E-01
IL 4 sR	0.04	0.23	8.75E-01	0.069	3.62E-01
calreticulin	0.01	0.08	8.75E-01	0.026	7.32E-01
DLL4	-0.01	0.09	8.79E-01	-0.007	9.32E-01
KLRF1	-0.02	0.10	8.80E-01	0.047	5.35E-01

CLF 1 CLC Complex	0.02	0.13	8.81E-01	-0.067	3.73E-01
PKC G	0.01	0.09	8.82E-01	0.005	9.48E-01
GV	0.02	0.13	8.83E-01	0.006	9.41E-01
IL 11 RA	0.03	0.20	8.85E-01	-0.093	2.19E-01
Mesothelin	-0.01	0.07	8.89E-01	-0.006	9.41E-01
BMX	0.01	0.10	8.91E-01	-0.050	5.10E-01
ABL2	0.01	0.10	8.92E-01	-0.020	7.91E-01
Granulysin	0.02	0.13	8.92E-01	0.022	7.68E-01
ASGR1	0.01	0.10	8.92E-01	-0.065	3.94E-01
b2 Microglobulin	-0.06	0.44	8.92E-01	0.102	1.81E-01
TNF b	0.01	0.09	8.93E-01	0.025	7.39E-01
IFN g R1	0.02	0.14	8.93E-01	-0.037	6.40E-01
Flt3 ligand	0.01	0.11	8.98E-01	0.066	3.83E-01
IL 18 Rb	0.02	0.12	8.99E-01	0.023	7.57E-01
IL 12 Rb1	0.03	0.22	9.00E-01	0.118	1.20E-01
BASI	-0.01	0.08	9.02E-01	-0.137	7.04E-02
Fucosyltransferase 3	-0.02	0.14	9.02E-01	-0.075	3.21E-01
IL 1 sRI	-0.02	0.13	9.04E-01	-0.040	6.01E-01
Desmoglein 1	0.01	0.12	9.05E-01	0.001	9.87E-01
PlGF	-0.01	0.10	9.06E-01	0.059	4.38E-01
TXD12	-0.01	0.11	9.07E-01	0.076	3.18E-01
ICOS	-0.03	0.22	9.08E-01	0.205	6.24E-03
FABP	-0.01	0.13	9.10E-01	-0.003	9.65E-01
IL 15 Ra	-0.04	0.34	9.11E-01	-0.032	6.69E-01
CYTD	0.02	0.21	9.11E-01	-0.036	6.33E-01
sLeptin R	-0.01	0.12	9.12E-01	-0.023	7.66E-01
HCC 4	-0.01	0.09	9.13E-01	-0.033	6.60E-01
PKC D	0.01	0.12	9.15E-01	-0.070	3.60E-01
LIMP II	-0.01	0.08	9.15E-01	0.002	9.83E-01
FN1 4	-0.01	0.10	9.17E-01	-0.026	7.30E-01
dopa decarboxylase	0.01	0.14	9.20E-01	0.004	9.58E-01
ADAM12	0.01	0.09	9.20E-01	0.125	9.86E-02
Activated Protein C	0.01	0.10	9.20E-01	0.184	1.44E-02
CK BB	0.01	0.09	9.22E-01	0.022	7.67E-01
IL 3	-0.01	0.10	9.23E-01	-0.029	6.98E-01
PLCG1	0.01	0.09	9.23E-01	-0.017	8.25E-01
Kallikrein 11	-0.01	0.10	9.25E-01	0.086	2.58E-01
BAFF Receptor	0.02	0.23	9.25E-01	0.040	5.98E-01
TBK1	0.01	0.09	9.26E-01	-0.109	1.48E-01
DAN	0.03	0.28	9.27E-01	0.095	2.09E-01
C3d	-0.01	0.12	9.30E-01	0.029	7.07E-01
IL 22BP	-0.01	0.11	9.30E-01	0.118	1.19E-01
ATP synthase beta chain	0.01	0.10	9.32E-01	0.041	5.88E-01
bFGF R	-0.01	0.10	9.32E-01	0.039	6.05E-01
Coagulation Factor VII	-0.01	0.13	9.32E-01	-0.055	4.68E-01

IGF I	-0.01	0.11	9.34E-01	0.112	1.39E-01
TCCR	0.01	0.13	9.35E-01	0.035	6.44E-01
KI3S1	0.01	0.16	9.37E-01	0.125	9.87E-02
EphA5	-0.01	0.13	9.39E-01	0.107	1.58E-01
IL24	-0.01	0.11	9.39E-01	-0.009	9.01E-01
GHC2	0.01	0.12	9.40E-01	0.048	5.26E-01
Carbonic anhydrase 9	0.01	0.11	9.40E-01	-0.009	9.08E-01
ANGL4	-0.01	0.12	9.40E-01	0.139	6.56E-02
Cardiotrophin 1	0.01	0.09	9.42E-01	-0.024	7.54E-01
Endoglin	-0.01	0.13	9.43E-01	0.057	4.48E-01
SPTA2	-0.01	0.10	9.44E-01	0.059	4.32E-01
PHI	-0.01	0.10	9.44E-01	-0.027	7.27E-01
OLR1	0.01	0.21	9.45E-01	0.087	2.53E-01
Enterokinase	0.01	0.15	9.45E-01	-0.068	3.69E-01
GA733 1 protein	0.01	0.17	9.48E-01	0.087	2.51E-01
PPase	-0.01	0.12	9.51E-01	-0.122	1.04E-01
MMP 9	0.01	0.11	9.51E-01	-0.032	6.70E-01
IL 13	0.01	0.10	9.54E-01	0.035	6.47E-01
sFRP 3	0.02	0.44	9.55E-01	0.048	5.24E-01
FGF 8B	-0.02	0.30	9.55E-01	0.016	8.34E-01
Discoidin domain receptor 2	-0.01	0.13	9.58E-01	0.017	8.23E-01
FCAR	-0.01	0.13	9.59E-01	0.031	6.80E-01
MATN2	0.00	0.08	9.59E-01	0.054	4.75E-01
Cytochrome c	-0.02	0.35	9.60E-01	0.009	9.09E-01
FAM107B	-0.01	0.15	9.63E-01	-0.093	2.20E-01
IMDH2	0.00	0.09	9.63E-01	0.077	3.08E-01
Semaphorin 3A	0.00	0.08	9.67E-01	0.082	2.79E-01
Galectin 2	0.00	0.10	9.68E-01	0.007	9.22E-01
XTP3A	-0.01	0.17	9.68E-01	0.096	2.06E-01
CD109	0.00	0.13	9.69E-01	0.005	9.51E-01
OX2G	0.00	0.11	9.70E-01	0.131	8.25E-02
carbonic anhydrase II	0.01	0.15	9.71E-01	0.027	7.18E-01
Periostin	0.00	0.10	9.72E-01	-0.115	1.30E-01
FCRL3	0.00	0.12	9.73E-01	-0.058	4.44E-01
TNF a	0.01	0.23	9.73E-01	0.022	7.71E-01
Heparin cofactor II	0.00	0.12	9.74E-01	0.079	2.95E-01
C3a	0.00	0.11	9.75E-01	0.125	9.95E-02
MDC	0.00	0.14	9.77E-01	0.089	2.38E-01
FGR	0.00	0.09	9.77E-01	-0.031	6.79E-01
MICB	0.00	0.13	9.77E-01	-0.034	6.57E-01
PFD5	-0.01	0.18	9.77E-01	0.040	5.98E-01
TSLP R	0.00	0.11	9.78E-01	0.034	6.55E-01
MMP 14	0.01	0.26	9.79E-01	-0.043	5.75E-01
B7 2	0.00	0.18	9.79E-01	0.030	6.90E-01
TrkC	0.00	0.14	9.83E-01	0.102	1.78E-01

Secretin	0.00	0.09	9.84E-01	-0.078	3.02E-01
FCG3B	0.01	0.40	9.87E-01	-0.005	9.45E-01
KI3L2	0.00	0.09	9.87E-01	-0.030	6.97E-01
C1r	0.00	0.13	9.90E-01	0.070	3.55E-01
DPP2	0.00	0.24	9.91E-01	0.035	6.47E-01
Siglec 6	0.00	0.11	9.93E-01	0.022	7.67E-01
MIP 3a	0.00	0.11	9.95E-01	0.057	4.49E-01
GFAP	0.00	0.19	9.97E-01	0.028	7.13E-01
ER	0.00	0.10	9.97E-01	-0.074	3.30E-01
IL 12	0.00	0.12	9.97E-01	-0.026	7.30E-01
PDE5A	0.00	0.09	9.98E-01	-0.023	7.66E-01
MMP 3	0.00	0.08	9.98E-01	0.172	2.23E-02
HINT1	0.00	0.08	9.98E-01	0.053	4.86E-01
BMP 7	0.00	0.14	9.99E-01	0.011	8.82E-01
Dynactin subunit 2	0.00	0.20	9.99E-01	-0.023	7.60E-01
GIB	0.00	0.12	1.00E+00	0.047	5.37E-01

Table S5 – List of significant enriched biological process of the 10 identified proteins using the PANTHER classification system of the gene ontology (GO) database. Statistics are provided by the PANTHER overrepresentation test. Bonferroni correction was applied to correct for multiple testing.

**Gene ontology biological process**	**Annotation Data Set (GO Ontology database Released 2017-08-14)**			**Fold Enrichment**	**P value after Bonferroni correctoin**
	**All reference annotations**	**Number of observed annotations**	**Number of expected annotations**		
Regulation of fibrinolysis	14	3	0.01	> 100	4.19E-04
Negative regulation of response to external stimulus	263	5	0.14	36.3	1.15E-03
Regulation of cellular protein metabolic process	2510	9	1.31	6.85	1.87E-03
Regulation of multicellular organismal process	2749	9	1.44	6.25	4.15E-03
Regulation of protein metabolic process	2750	9	1.44	6.25	4.17E-03
Regulation of response to external stimulus	763	6	0.4	15.01	7.80E-03
Negative regulation of response to stimulus	1421	7	0.74	9.41	1.44E-02
Negative regulation of hemostasis	46	3	0.02	> 100	1.47E-02
Negative regulation of blood coagulation	46	3	0.02	> 100	1.47E-02
Positive regulation of fibrinolysis	4	2	0	> 100	1.71E-02
Negative regulation of coagulation	50	3	0.03	> 100	1.89E-02
Negative regulation of wound healing	65	3	0.03	88.12	4.13E-02
Negative regulation of multicellular organismal process	1031	6	0.54	11.11	4.49E-02
Response to wounding	558	5	0.29	17.11	4.60E-02
Acute inflammatory response	69	3	0.04	83.01	4.93E-02
